# Whole-Genome Sequencing and Genome-Wide Studies of Spiny Head Croaker (*Collichthys lucidus*) Reveals Potential Insights for Well-Developed Otoliths in the Family Sciaenidae

**DOI:** 10.3389/fgene.2021.730255

**Published:** 2021-09-30

**Authors:** Wu Gan, Chenxi Zhao, Xinran Liu, Chao Bian, Qiong Shi, Xinxin You, Wei Song

**Affiliations:** ^1^ East China Sea Fisheries Research Institute, Chinese Academy of Fishery Sciences, Shanghai, China; ^2^ Key Laboratory of Exploration and Utilization of Aquatic Genetic Resources, Ministry of Education, Shanghai Ocean University, Shanghai, China; ^3^ BGI Education Center, University of Chinese Academy of Sciences, Shenzhen, China; ^4^ Shenzhen Key Lab of Marine Genomics, Guangdong Provincial Key Lab of Molecular Breeding in Marine Economic Animals, BGI Academy of Marine Sciences, BGI Marine, BGI, Shenzhen, China

**Keywords:** spiny head croaker, whole genome sequencing, chromosome-level genome assembly, otolith development, Sciaenidae

## Abstract

Spiny head croaker (*Collichthys lucidus*), belonging to the family Sciaenidae, is a small economic fish with a main distribution in the coastal waters of Northwestern Pacific. Here, we constructed a nonredundant chromosome-level genome assembly of spiny head croaker and also made genome-wide investigations on genome evolution and gene families related to otolith development. A primary genome assembly of 811.23 Mb, with a contig N50 of 74.92 kb, was generated by a combination of 49.12-Gb Illumina clean reads and 35.24 Gb of PacBio long reads. Contigs of this draft assembly were further anchored into chromosomes by integration with additional 185.33-Gb Hi-C data, resulting in a high-quality chromosome-level genome assembly of 817.24 Mb, with an improved scaffold N50 of 26.58 Mb. Based on our phylogenetic analysis, we observed that *C. lucidus* is much closer to *Larimichthys crocea* than *Miichthys miiuy*. We also predicted that many gene families were significantly expanded (*p*-value <0.05) in spiny head croaker; among them, some are associated with “calcium signaling pathway” and potential “inner ear functions.” In addition, we identified some otolith-related genes (such as otol1a that encodes Otolin-1a) with critical deletions or mutations, suggesting possible molecular mechanisms for well-developed otoliths in the family Sciaenidae.

## Introduction

Spiny head croaker (*Collichthys lucidus*), belonging to the family Sciaenidae, is a small economic fish with a main distribution in the coastal waters of Northwestern Pacific (Cheng et al., 2012a), from Philippines, China to Japan. With excellent properties and good meat quality, spiny head croaker has been favored by Chinese consumers with a high market value, whereas it has been overfished in the Yangtze estuary area of China (Hu et al., 2015). Furthermore, Sciaenidae fishes are well known for their well-developed otoliths (Xu et al., 2016), which are acellular crystalline mineral deposits in the inner ears of various teleost fishes (Pracheil et al., 2019).

Otoliths, mainly composed of calcium carbonate and organic matrix, play vital roles in sound sensing, balance, linear acceleration, and gravity in bony fishes (Schulz-Mirbach et al., 2019). Moreover, otoliths are widely applied in fisheries sciences, such as evaluation of fish populations or population migration patterns, and are essential for paleoichthyological and archeological studies (Barrett, 2019; Barnett et al., 2020; Heimbrand et al., 2020). Several critical genes, including Otoconin-90 (Oc90), secreted protein acidic and rich in cysteine (SPARC), SPARC-like1 (SPARCL1), otopetrin-1 (otop1), otolin-1 (otol1), and Otolithmatrixprotein-1 (OMP-1), have been identified to be related to otolith growth and formation in various vertebrates including bony fishes (Hurle et al., 2003; Murayama et al., 2005; Kang et al., 2008; Petko et al., 2008; Xu et al., 2016b; Hołubowicz et al., 2017). Nevertheless, a systematic screening of otolith-related genes in Sciaenidae species has not been reported yet.

With the rapid development of genome sequencing technology and genome-based bioinformatics methods, studies on aquatic genomes and related applications, such as molecular breeding, drug development, new biomaterials, and DNA barcoding technology, have been accumulated (Zhang et al., 2019; Houston et al., 2020; Li et al., 2020; Long et al., 2020). Whole-genome sequencing (WGS) of about 90 fishes has been published around the world by far (Bian et al., 2019; You et al., 2020). At present, genome studies on the Sciaenidae family have focused on the popular large yellow croaker (*Larimichthys crocea*) and its economic traits (Wu et al., 2014; Ao et al., 2015; Gui et al., 2018; Mu et al., 2018; Chen et al., 2019), while there are only few reports on other Sciaenidae species such as spiny head croaker.

Mitochondrial genome maps of spiny head croaker and candidate genes related to its sex determination have been examined by mitochondrial genome sequencing (Cheng et al., 2012b), chromosome assembly (Cai et al., 2019), and RNA sequencing (Song et al., 2020). However, the genetic basis of well-developed otoliths in Sciaenidae species is still unknown. It is therefore necessary to explore genomic resources to gain insights into otolith development mechanisms and to accelerate genome-assisted improvements in biodiversity protection, breeding, and disease prevention of Sciaenidae species.

In the past decades, Illumina short-read sequencing technology has been generally employed to assemble various fish genomes. However, with cost reduction of both short- and long-read sequencing technologies, more and more recent WGS projects have introduced PacBio long-read sequences in order to assemble high repetitive regions and improve assembly quality (You et al., 2020). Here, we produced a nonredundant chromosome-level assembly of the spiny head croaker by combination of Illumina short reads, PacBio Single Molecule Real-Time (SMRT) long reads, high-throughput chromosome conformation capture (Hi-C) data, and transcriptome sequences. Moreover, we performed comparative genomics studies on candidate genes related to otolith development to figure out potential mechanisms for the well-developed otoliths in the family Sciaenidae.

## Materials and Method

### Sample Collection and Sequencing

We extracted genomic DNAs from pooled muscle tissues of a wild female spiny head croaker and sequenced by using an Illumina HiSeq 2500 sequencing platform (San Diego, CA, United States) and a PacBio Sequel sequencing platform (Menlo Park, CA, United States). The construction of DNA libraries (insert sizes of 500 and 800 bp for Illumina, and 20 kb for PacBio) and subsequent sequencing were performed according to the standard protocols. In total, 52.48 Gb of raw Illumina data and five SMRT cells produced using the P6 polymerase/C4 chemistry, producing 35.24 Gb of PacBio long reads, were generated. After filtering by SOAPnuke (v.1.5.6; Chen et al., 2017), we obtained 49.12 Gb of Illumina clean data and 35.24 Gb of PacBio data for subsequent assembly.

To acquire a chromosome-level assembly of the genome, genomic DNAs were fixed with formaldehyde and were sheared by a restriction enzyme (MboI) to build a Hi-C library, and then sequenced by an Illumina HiSeq X Ten platform. A total of 185.33 Gb of 150 PE Hi-C data were generated. All sequenced data generated in this study were deposited in the CNGB Nucleotide Sequence Archive under Program no. CNP0001197.

We also extracted muscle RNA for transcriptome sequencing by using a HiSeq 2500 platform. Furthermore, to obtain the full-length transcript, the mixed RNA sample from 13 tissues was transcribed to generate full-length cDNA, and the SMRT bell library was constructed using the SMRT bell Template Prep Kit. The libraries were then prepared for sequencing on the PacBio Sequel sequencing platform.

### Genome Assembly and Chromosome Assembly

Firstly, we employed Kmerfreq (https://github.com/fanagislab/kmerfreq) to estimate the genome size with 17-bp k-mers and applied GenomeScope (v1.0; Vurture et al., 2017) to estimate genome heterozygosity. Subsequently, a hybrid genome assembly pipeline was employed to obtain genome assembly. Short Illumina reads were first assembled by using Platanus with “-m 300 -k 27 -s 3” (Kajitani et al., 2014), and DBG2OLC (Ye et al., 2016) was performed to combine Platanus-generated contigs with PacBio reads to generate a hybrid contig assembly with default parameters. Pilon (v.1.225; (Walker et al., 2014) was employed to polish the hybrid assembly. After then, redundancies of the primary assembly were removed by Redundans (Pryszcz and Gabaldón, 2016) with “--identity 0.85 --overlap 0.36.”

We performed quality control of Hi-C raw reads and obtained valid Hi-C-connected reads by Juicer (v.1.5; Durand et al., 2016). A 3D *de novo* assembly (3D-DNA, v.180922; Dudchenko, et al., 2017) pipeline (Dudchenko et al., 2017) was applied to anchor primary contigs into chromosome-level scaffolds. Completeness of the genome assembly was evaluated using by BUSCO v3.0 (Simão et al., 2015) with “-l actinopterygii_odb9 -m genome -c 3 -sp zebrafish.”

### *De Novo* Assembly of Transcriptomes

We *de novo* assembled the RNA-seq reads using the Trinity assembler (v2.9.0; Haas et al., 2013) and TGI clustering tool (TGICL; Pertea et al., 2003). The PacBio ISO-Seq3 pipeline (https://github.com/PacificBiosciences/IsoSeq) was used to obtain full-length non-chimeric (FLNC) transcripts *via* ccs, classify, cluster, and polish stage. FLNC was aligned with genome by minimap2.

### Gene Prediction and Annotation

Repetitive elements in the spiny head croaker genome were identified through a combination of homolog-based and *de novo* approaches. For the homolog-based method, RepeatMasker (v.4.0.7) (Smit et al., 2019) and RepeatProteinMask (v.4.0.7; Smit et al., 2019) were used to detect repeats by aligning against the Repbase database (v 21.0; Bao et al., 2015). For the *de novo* method, LTRharvest (Ellinghaus et al., 2008) was applied to predict full long terminal repeat (LTR) retrotransposons. RepeatModeler (v1.0.11; Smit et al., 2019) was employed to build transposable element (TE) consensus sequences as a *de novo* TE library, and TRF (v.4.09; Benson, 1999) was used to obtain tandem repetitive sequences. RepeatMasker was then used to discover and identify repetitive sequences with the combined library of the *de novo* TEs.

Based on the repeat masked genome, we employed *de novo*, homology-based, and transcriptome-based prediction methods to annotate protein-coding genes in the assembled genome. Protein sequences of zebrafish (*Danio rerio*), three-spined stickleback (*Gasterosteus aculeatus*), Atlantic cod (*Gadus morhua*), channel catfish (*Ictalurus punctatus*), spotted gar (*Lepisosteus oculatus*), Nile tilapia (*Oreochromis niloticus*), fugu (*Takifugu rubripes*), downloaded from Ensembl (Hunt et al., 2018), and large yellow croaker (*L. crocea*, GCF_000972845.2) from NCBI were aligned to the spiny head croaker genome by tBLASTn (Kent, 2002) with “-e 1e-5.” Subsequently, GeneWise (Birney et al., 2004) was used to predict gene structures from BLAST hits. Augustus (v3.3.1; Stanke et al., 2006) was performed to predict *de novo* genes. We obtain 3,000 intact gene models generated from the homolog-based method randomly to train the parameters of AUGUSTUS then used AUGUSTUS to perform *de novo* prediction based on the repeat-masked genome with the training parameters. These gene sets that were predicted by different methods were integrated into a nonredundant gene set through the pipelines described in previous research (Xiong et al., 2016). After that, the combined gene set was modified with transcriptome data through PASA (v2.3.3; Haas et al., 2003).

Gene functional annotation was performed based on consensus of sequence and domain. The protein sequences were aligned to NCBI Non-Redundant Protein Sequence (NR) databases, Kyoto Encyclopedia of Genes and Genomes (KEGG v89.0; Kanehisa and Goto, 2000), SwissProt, and TrEMBL (Uniprot release 2020-06) (Boeckmann et al., 2003) by BLASTp with “-e 1e-5.” The domains were searched and predicted by using InterProScan v5.11–55.0 (Zdobnov and Apweiler, 2001) (Jones et al., 2014) with publicly available databases including PANTHER (Thomas et al., 2003), Pfam (Bateman et al., 2004), PRINTS (Attwood et al., 2000), ProDom (Servant et al., 2002), PROSITE profiles (Sigrist et al., 2010), and SMART (Letunic et al., 2012). Gene ontology (GO) terms (Ashburner et al., 2000) for each gene were predicted from the InterPro descriptions.

### Genome Evolution and Gene Family Analysis

In order to identify gene families in spiny head croaker, we collected protein sequences of the same species used for homologous annotation as well as miiuy croaker (*Miichthys miiuy*) and performed the TreeFam methodology (Li et al., 2006) to obtain gene families of these species. We then used RaxML (Stamatakis, 2006) to construct the phylogenetic tree by using the single copy orthologous gene families with the GTRGAMMA model.

To identify the synteny between spiny head croaker and large yellow croaker, BLASTp was used to calculate pairwise similarities (e value < 1e-5), and MCScanX package with default parameters was then used for classification. Then, JCVI was performed to generate visualization.

A MCMCtree program in PAML (v4.9e; Yang, 2007) was performed to estimate the divergence time between various species in the phylogenetic tree with the REV substitution model. Three calibration time points based on the TimeTree database (http://www.timetree.org) were used as references (*T. rubripes*-*G. aculeatus*: 99–127 MYA; *G. morhua*-*T. rubripes:* 141–166 MYA; *L. oculatus*-*D. rerio*: 295–334 MYA), including spotted gar, zebrafish, Atlantic cod, fugu, and three-spined stickleback.

CAFÉ (v3.0; Han et al., 2013) was used to analyze gene family expansion and contraction under a maximum likelihood framework; single-copy orthologous gene families and estimated divergence time between different species were used as input files. To identify possible positive selected genes (PSGs), we first conducted multiple-sequence alignments based on the protein sequences of single-copy gene families by PRANK (Löytynoja, 2014), then the non-synonymous substitution rate (Ka) and synonymous substitution rate (Ks) were calculated by the codeml in PAML (v4.9e) with the branch-site model (cleandata = 1) and spiny head croaker was chosen as foreground species. Only the results with *p*-values <0.05 and false discovery rate (FDR) < 0.05 were considered as positive selected genes. Based on whole-genome annotation results and the official classification, we use the phyper in R (v3.5.2) to perform KEGG pathway enrichment analysis.

### Phylogenetic Analysis of Otolith Related Genes

We downloaded the protein sequences of Otolin-1a, Otolin-1b, Otopetrin-1, Otoconin-90, OMP-1, SPARC, SPARCL1, and SPARCL2 from zebrafish and fugu and whole-genome sequences of four fishes (*D. rerio*, *L. crocea*, *T. rubripes*, and *Dicentrarchus labrax*) from NCBI and Ensembl. Nucleotide sequences of these genes were aligned from these four species and spiny head croaker (from the present study) by using BLAST, and filtered with identity, then related protein coding sequences were predicted by Exonerate (v.2.2.0) (Slater and Birney, 2005) or GeneWise firstly. Secondly, we converted coding sequences (CDS) to protein sequences and used PRANK (Löytynoja, 2014) to perform multiple-sequence alignments. RaxML (Stamatakis, 2006) was employed to construct a gene family phylogenetic tree with the PROTGAMMAAUTO model, and the genes from spotted gar as outgroup. We also predicted whether these protein-coding genes in Sciaenidae were involved in positive selection by using the codeml in PAML (v4.9e) with the branch-site model (cleandata = 1) and choosing the branch of spiny head croaker and large yellow croaker as foreground species, and searched the domains of these protein sequences by using NCBI Batch CD-Search and generated visualizations *via* EvolView (Subramanian et al., 2019).

According to the results of multiple-sequence alignments and PSG analysis, we selected those amino acid sites with inconsistency between the family Sciaenidae and other species. Potential functional effects of these residual substitutions were evaluated by PolyPhen-2 (Adzhubei et al., 2013; Li et al., 2018) and PROVEAN (Choi and Chan, 2015).

## Results

### Genome Sequencing and Assembly

We sequenced the genome of a wild female spiny head croaker by using an Illumina HiSeq sequencing platform as well as a PacBio Sequel sequencing platform. After data filtering, we obtained a total of 49.12-Gb Illumina clean reads by SOAPnuke and 35.24-Gb PacBio long reads, representing approximately 60-fold and 43-fold coverage of the spiny head croaker genome, respectively. An entire genome size of 811.25 Mb was estimated by the routine Kmerfreq method (with K = 17; https://github.com/fanagislab/kmerfreq). Employing a hybrid assembly method, we obtained a redundant assembly of 994.29 Mb and then used Redundans (Kajitani et al., 2014; Ye, et al., 2016) to reduce the redundant sequences. About 18.4% sequences in hybrid assembly were removed. We obtained a draft genome of 811.23 Mb with a contig N50 of 74.92 kb (Table 1, Supplementary Table S1). The mapping ratio with genome sequencing was 97.89% for the chromosome version (Supplementary Table S2).

**TABLE 1:** Statistics of the genome assembly of spiny head croaker.

Parameter	Contig	Scaffold
Total length (bp)	811,227,110	817,240,112
Total number	12,220	10,973
Gap (bp)	0	6,013,002
N50 (bp)	74,891	26,576,940
N90 (bp)	38,245	23,883
Maximum length (bp)	428,343	33,752,147
Hi-C anchored length (bp)	0	643,229,385
Hi-C anchored rate	0	78.71%
GC content	41.60%	41.30%
Evaluation of BUSCO	94.00%	94.20%

A total of 185.33 Gb Hi-C data were analyzed by Juicer, and contigs in the draft assembly were subsequently anchored into chromosomes by a 3D-DNA pipelin, resulting in a polished genome assembly of 817.24 Mb, with an improved scaffold N50 of 26.58 Mb (Supplementary Figure S1, Table 1). The final assembly consists of 24 chromosomes (ranging from 24.92 to 33.75 Mb in length) and covers 643.23 Mb which accounts for 78.71% of the whole genome (Supplementary Table S3).

We determined that approximately 94.2% of complete reference genes (82.3% single-copy and 11.9% duplicated) were detectable in the final assembly according to BUSCO values (Supplementary Table S4).

### Gene Prediction and Annotation

**FIGURE 1:**
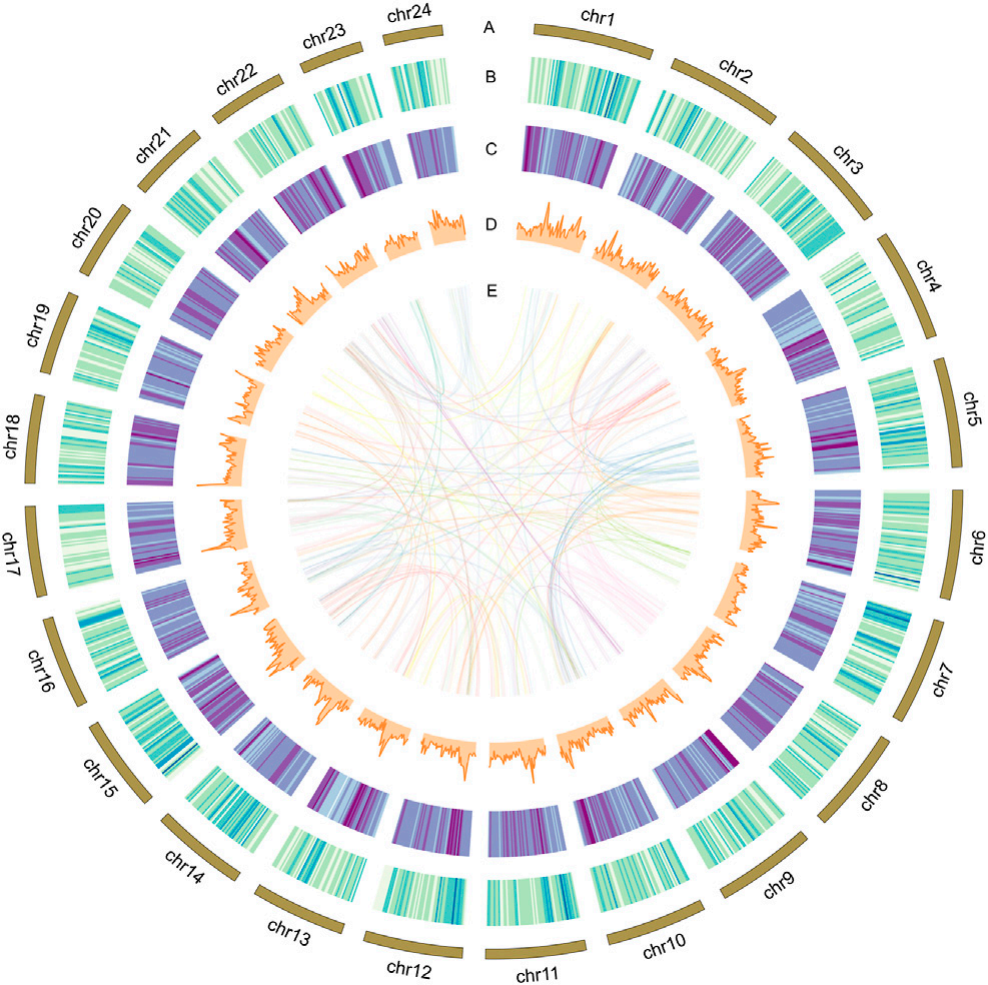
Characterization of the assembled genome for spiny head croaker. From outside to inside: **(A)** chromosomes of spiny head croaker; **(B)** gene density of the genome; **(C)** repeat density of the genome; **(D)** GC content of the genome; **(E)** paralogous genes on different chromosomes. **(B–D)** were drawn in 500-kb sliding windows.

In total, approximately 31.69% of the spiny head croaker assembled sequences were annotated as repetitive elements, which is higher than that for large yellow croaker (Ao et al., 2015; Mu et al., 2018). The repetitive sequences include 161.45 Mb of DNA transposons (∼19.90%), 76.60 Mb of long interspersed elements (LINEs, ∼9.44%), 73.46 Mb of long terminal repeats (LTRs, ∼9.06%), and other TEs (Table 2, Figure 1).

**TABLE 2:** Repetitive elements in the assembled genome of spiny head croaker.

Parameter	RepBase TEs		TE proteins		*De novo*		Combined TEs	
	Length (bp)	% in genome	Length (bp)	% in genome	Length (bp)	% in genome	Length (bp)	% in genome
DNA	54,209,863	6.682452	4,882,493	0.601865	138,693,323	17.09673	161,446,484	19.90152
LINE	24,524,858	3.02318	14,324,110	1.765734	51,652,425	6.367197	76,599,297	9.442399
SINE	4,234,508	0.521988	0	0	2,415,507	0.29776	6,410,232	0.79019
LTR	19,821,582	2.443407	9,298,472	1.146223	54,523,316	6.721091	73,457,448	9.055103
Other	9,297	0.001146	0	0	0	0	9,297	0.001146
Unknown	0	0	0	0	40,564,762	5.00042	40,564,762	5.00042
Total	94,358,167	11.631535	28,439,883	3.505786	219,148,467	27.01444	257,046,838	31.68617

Using the repeat-masked genome assembly, we predicted a total of 29,509 genes after integration of *de novo*, homology-based, and transcriptome-based annotations (Table 3). The total annotated gene number is similar to that in a previous published genome of the spiny head croaker (Figure 1, Supplementary Figure S2). Based on functional annotation, we predicted 29,432 (∼99.74%) protein-coding genes with at least one assignment from Swiss-Prot, TrEMBL, InterProScan, Nr database, and KEGGor GO databases.

**TABLE 3:** Predicted protein-coding genes in the genome of spiny head croaker.

Evidence	Method/species	Numbers	Average gene length (bp)	Average CDS length (bp)	Average exon length (bp)	Average exon per gene	Average intron length (bp)
*De novo*	AUGUSTUS	40,824	5,815.97	1,092.66	170.20	6.42	871.46
Homolog	*Danio rerio*	26,993	7,176.52	1,404.30	175.12	8.02	822.34
	*Gadus morhua*	26,917	6,874.98	1,290.37	166.71	7.74	828.53
	*Gasterosteus aculeatus*	27,805	7,206.74	1,366.09	166.06	8.23	808.25
	*Ictalurus punctatus*	27,527	7,096.90	1,368.55	175.28	7.81	841.41
	*Larimichthys crocea*	31,465	7,875.63	1,539.67	173.39	8.88	804.08
	*Lepisosteus oculatus*	25,501	7,310.53	1,396.47	174.29	8.01	843.40
	*Oreochromis niloticus*	30,849	7,664.18	1,543.67	180.70	8.54	811.43
	*Takifugu rubripes*	28,381	7,038.13	1,340.72	172.83	7.76	843.14
Transcriptome	—	16,189	4,348.24	1,295.77	160.29	8.08	2,609.32
Total		29,509	6,425.15	1,336.49	174.59	7.65	764.65

### Genome Evolution and Gene Family Analysis

**FIGURE 2:**
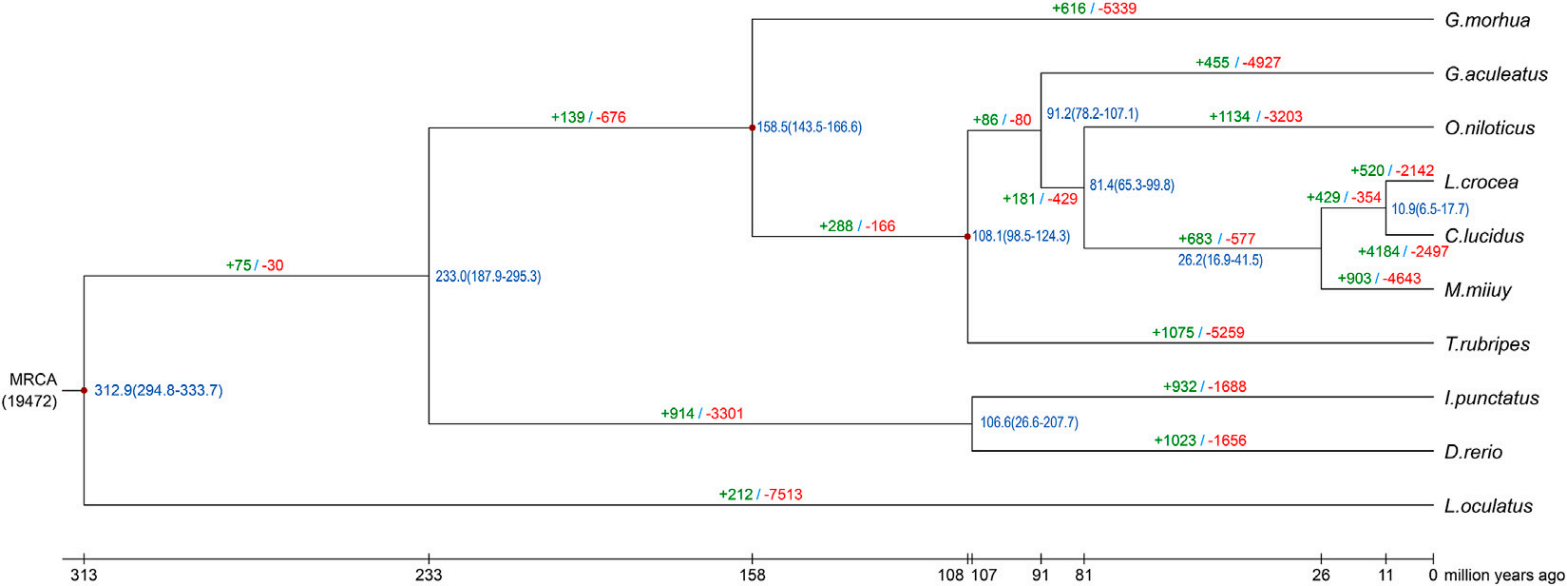
Evolution analysis of spiny head croaker. Green and red numbers on the branches represent the expansion and contraction gene families in each species, blue numbers on the branches show the estimated divergence times in Mya, and dark red points represent the calibration time from TimeTree.

To determine the phylogenetic relationship of spiny head croaker with other species, we compared its assembly with other nine representative fish genomes. We identified a total of 19,627 gene families (16,005 in spiny head croaker) and 3,955 single-copy orthologues from TreeFam. After construction of a phylogenetic tree by using the single-copy orthologous gene families, we observed that spiny head croaker is much closer to large yellow croaker (Figure 2, Supplementary Figure S3), and Cichlidae (such as Nile tilapia) is closely related to Sciaenidae (such as spiny head croaker, large yellow croaker, and miiuy croaker). According to the results of MCMCtree, we estimated that spiny head croaker and large yellow croaker diverged around 13 (5.8∼28.6) million years ago (Mya), and their ancestor diverged from tilapia around 81 (67.8∼97.4) Mya (Figure 2).

**FIGURE 3:**
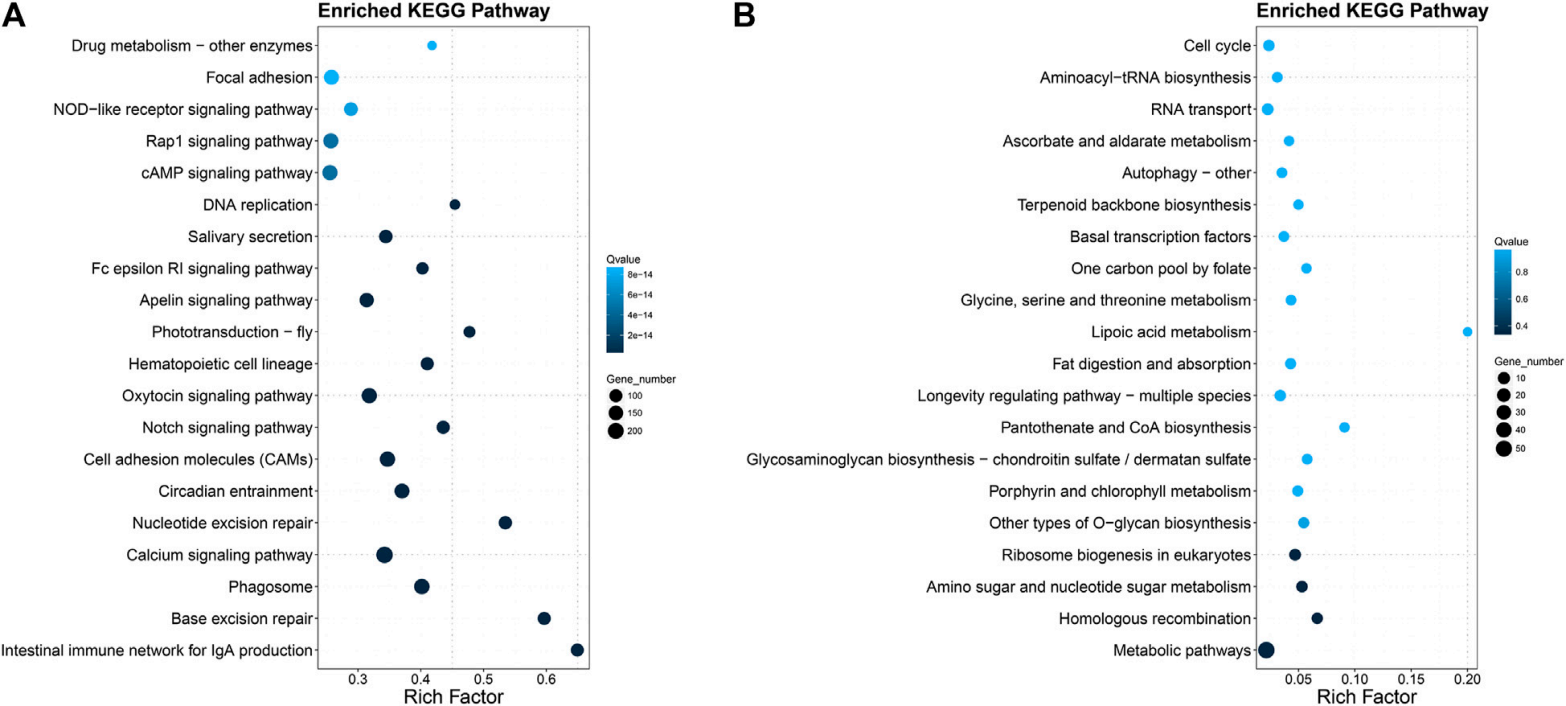
KEGG pathway enrichments in spiny head croaker. **(A)** Pathway enrichments of expanded gene families; **(B)** pathway enrichments of positive selected genes.

Based on the phylogenetic tree and species divergence time analysis, we employed CAFÉ to analyze the expansion and contraction of gene families. A total of 1,028 significantly expanded (*p* < 0.05) and 230 significantly contracted (*p* < 0.05) gene families were predicted in spiny head croaker (Figure 2). Interestingly, many expanded gene families were enriched in several important KEGG pathways (Figure 3A), such as “calcium signaling pathway” (*p* = 1.30e−39), “circadian entrainment” (*p* = 1.39e−32), “intestinal immune network for IgA production” (*p* = 4.40e−47), and “NOD-like receptor signaling pathway” (*p* = 5.68e−15).

According to the analysis of positive selection with single-copy gene families, 421 positive selected genes (PSGs; *p* < 0.05) were identified in spiny head croaker (Supplementary Table S5) by PAML (Yang, 2007). These PSGs were enriched in several interesting KEGG pathways, such as “amino sugar and nucleotide sugar metabolism,” “longevity regulating pathway,” and “fat digestion and absorption” (Figure 3B).

### Phylogenetic Analysis of Otolith-Related Genes

We examined several critical otolith-related genes, including otol1a, otol1b, otop1, oc90, omp1, sparc, sparcl1, and sparcl2, to find genetic evidence for the well-developed otoliths in the family Sciaenidae. All sequences were derived from five representative fishes, including zebrafish, fugu, and three Perciformes species (large yellow croaker, spiny head croaker, and European sea bass), and the sequences of zebrafish and fugu (download from NCBI and Ensembl) were used as the queries (Supplementary Table S6).

**FIGURE 4:**
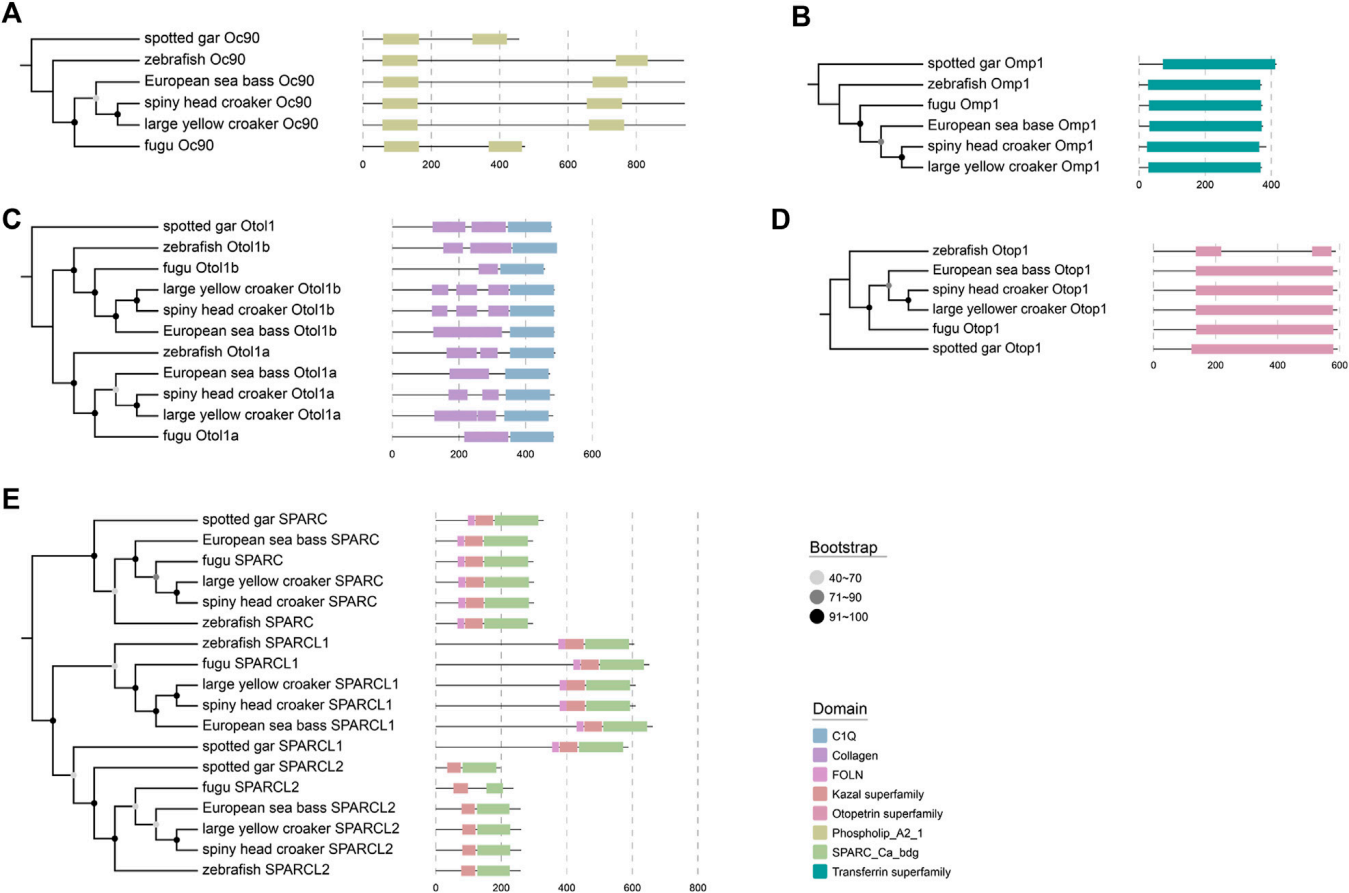
Phylogeny of otolith-related genes among six examined fish species. The detailed phylogenetic topology of Otoconin-90 [Oc90; **(A)**], Otolith matrix protein 1 [Omp-1; **(B)**], Otolin-1a (Otol1a) and Otolin-1b [Otol1b; **(C)**], Otopetrin-1 [Otop1; **(D)**], secreted protein acidic and rich in cysteine (SPARC), SPARC Like 1 (SPARCL1), and SPARC Like 2 [SPARCL2; **(E)**] was constructed individually.

Each of these genes was a single copy in these examined species. However, localization and multiple-sequence alignment displayed that the gene previously annotated as otol1 in the family Sciaenidae was more similar to zebrafish otol1b; another gene annotated as inner ear-specific collagen showed a higher sequence similarity to zebrafish otol1a. Domains of otolith-related genes were searched by NCBI Batch CD-Search, and our results proved that the examined domains of these genes were highly conserved in various species (see more details in Figure 4). Phylogenetic trees of these otolith-related genes were constructed, and their topological structures were consistent with the species tree. For example, large yellow croaker and spiny head croaker were clustered as sister groups (Figure 4), indicating that these genes had a closer relationship in these two croaker species than in other vertebrates.

**FIGURE 5:**
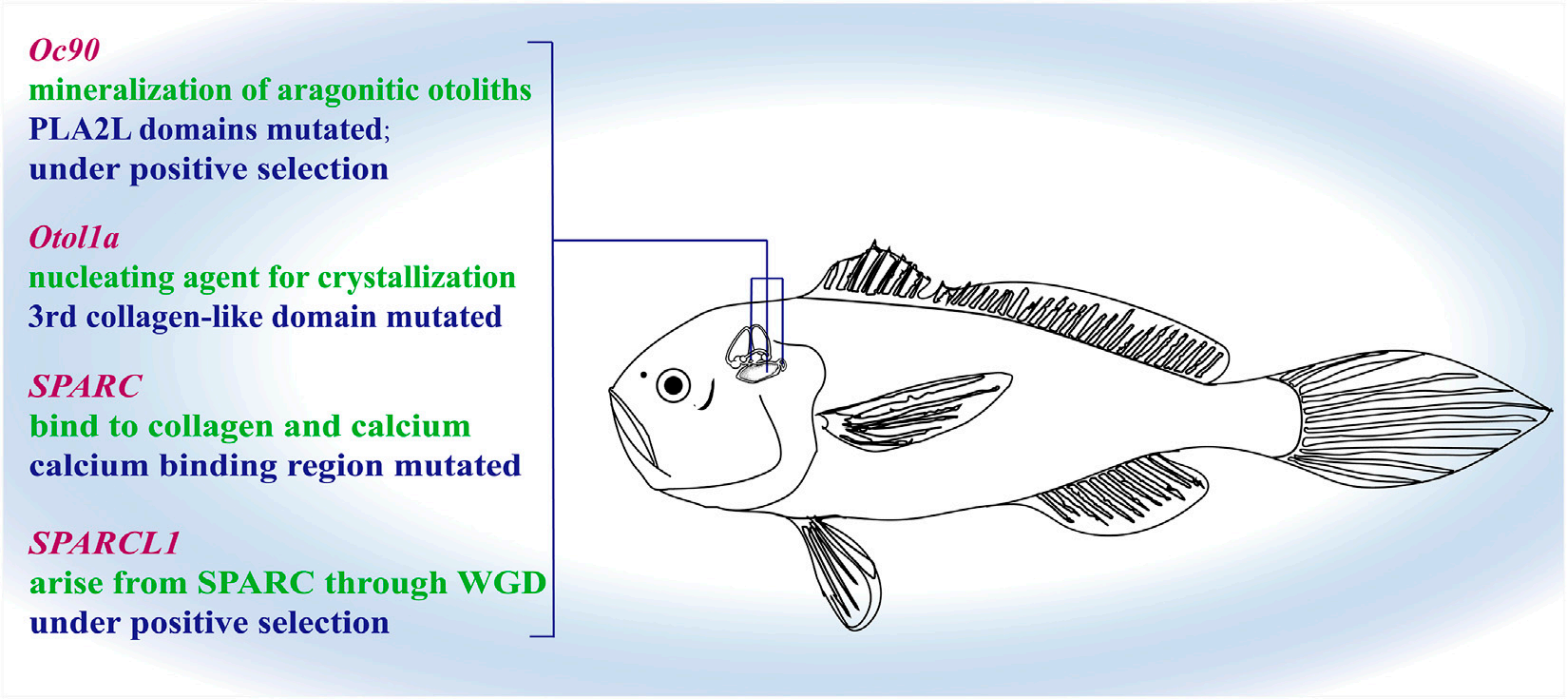
Genetic changes in candidate otolith related genes of the family Sciaenidae. We found that Oc90, Otol1a, and SPARC contained some mutated sites, and Otop1 and SPARCL1 were under positive selection in Sciaenidae.

**FIGURE 6:**
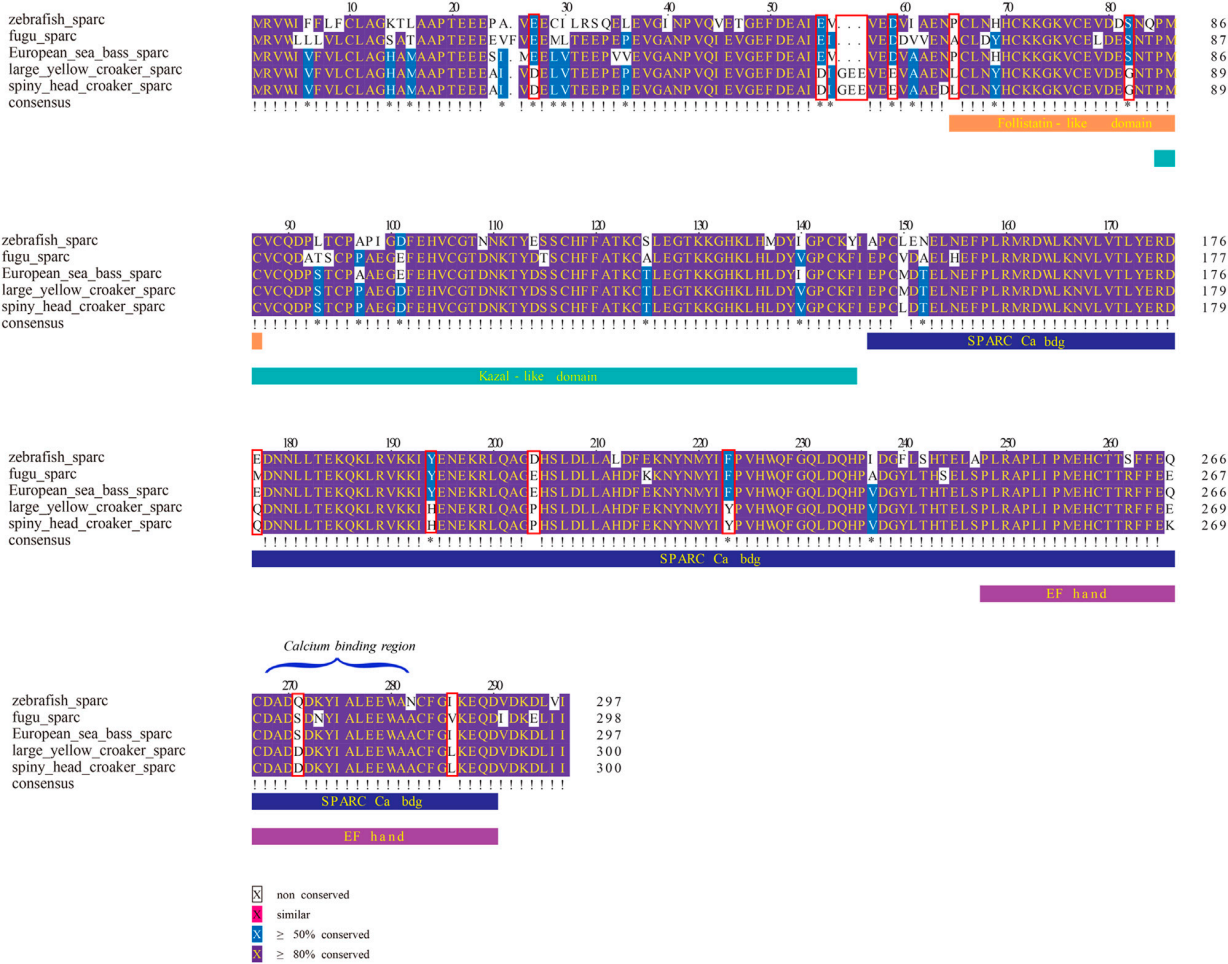
Multiple alignments of partial SPARC sequences. Red boxes mark the changed amino residues in SPARC from the family Sciaenidae.

**FIGURE 7:**
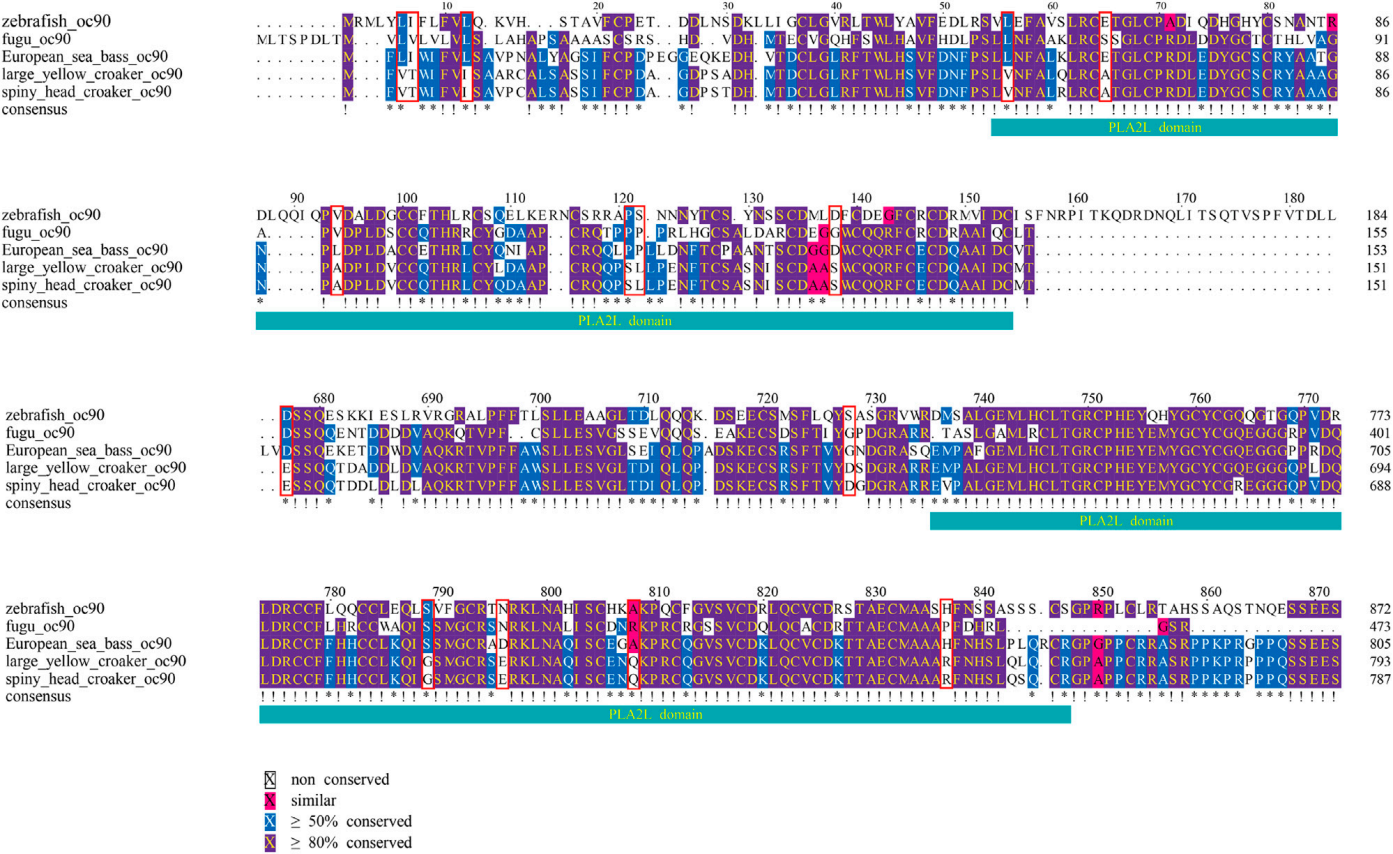
Multiple alignments of partial Oc90 sequences. Red boxes mark the changed amino residues at two PLA2L domains of the Oc90 from the family Sciaenidae.

**FIGURE 8:**
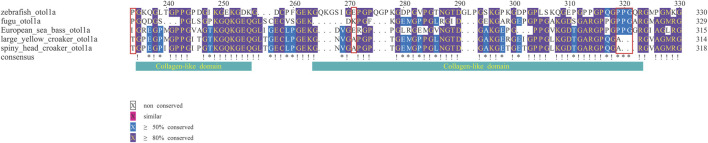
Multiple alignments of partial Otol1a sequences. Red boxes mark the changed amino residues at the collagen-like domain of Otol1a from the family Sciaenidae.

From the results of multiple-sequence alignment, we found that, in large yellow croaker and spiny head croaker, some nucleotide variances led to amino acid changes in some genes when compared with other fishes (see Figures 5–8, Supplementary Figures S4–S11). Interestingly, we observed that 16 amino acid residues in SPARC of the family Sciaenidae are different from those in other fishes, although the sequences of the calcium-binding region in SPARC showed high conservation in most species; however, the position 274 of large yellow croaker and spiny head croaker is Asp (D) instead of Gln (Q) or Ser (S) (see more details in Figure 6). In Oc90, several residues were changed at PLA2L domains (Figure 7).

Based on the best branch-site model, we propose that two critical otolith-related genes (SPARCL1 and OC90) in the family Sciaenidae were positively selected (Supplementary Table S7). Moreover, based on the multiple alignments, Some of the amino acid substitution sites in these genes were predicted to have a possible effect on the proteins by PolyPhen-2 analysis (Adzhubei et al., 2013) and by PROVEAN analysis (Choi and Chan, 2015), such as position 319 of Otol1a, positions 65 and 204 of SPARC, and the positive selected site in SPARCL1. More importantly, two deletion sites in Otol1a (positions 320 and 321) were predicted to have a possible effect on the protein by PROVEAN (Figure 8). Through PAML, we further predicted whether these genes were under positive selection or not.

## Discussion

Due to the high heterozygosity rate of some species, heterozygous regions are probably assembled repeatedly, resulting in a redundant genome with a larger size. According to GenomeScope, heterozygosity of spiny head croaker is about 1%. In the present study, we chose a hybrid assembly strategy with a combination of Illumina short reads and PacBio long reads and then employed Redundans (Pryszcz and Gabaldón, 2016) to remove the redundant region so as to obtain a final genome as much as possible to be equal to the estimated haploid genome size. Using this improved version of genome assembly, we reconstructed 24 chromosomes with additional Hi-C reads. The genome size of spiny head croaker (817.24 Mb) is bigger than that of large yellow croaker (708.47 Mb; Chen et al., 2019) and miiuy croaker (636.22 Mb; Xu et al., 2016a). According to our results, especially the annotation of repetitive sequences in this study, we speculate that the higher proportion of repetitive elements may be the major reason for the larger genome of spiny head croaker than its close relatives.

In our present study, a total of 29,509 genes were annotated from the spiny head croaker genome, which are more than those from a previous study (Cai et al., 2019). The number is higher than that of large yellow croaker (23,172 genes; Chen et al., 2019), miiuy croaker (21,960 genes; Xu et al., 2016a), and leopard coral grouper (25,248 genes; Zhou et al., 2020), whereas it is similar to Nile tilapia (29,249 genes; Conte et al., 2017). Moreover, our current work investigated gene families in spiny head croaker based on an integration of PacBio long-read sequencing and Hi-C technology. We identified 3,955 single-copy orthologues and observed that spiny head croaker is much close to large yellow croaker, which is consistent with previous mitochondrial genome studies (Cheng et al., 2012b). Further analysis of gene families showed that some gene families have significantly expanded in the spiny head croaker. Many gene families were enriched in several important pathways, such as “calcium signaling pathway” and “NOD-like receptor signaling pathway,” which may be related to some biological characteristics and basic physiological activities of this economically important fish.

Interestingly, in the “calcium signaling pathway,” we predicted that the calcium-binding protein (CaBP) gene family and parvalbumin gene family were significantly expanded in the spiny head croaker. Previous studies reported that CaBPs and parvalbumin are early markers of non-mitotic regenerating hair cells in bullfrog vestibular otolith (Steyger et al., 1997). CaBPs, located at the neuroretina, inner ear, and notochord, could modulate calcium levels and distribution, and thereby they were regarded as important regulators of essential neuronal target proteins (Haynes et al., 2012; Di Donato et al., 2013). Otolith-specific CaBPs were also detected in zebrafish (Söllner et al., 2003) and rainbow trout (Poznar et al., 2017). It was reported that the circadian rhythm of hair cells for secreting these CaBPs is likely to be a vital factor to cause the daily increase of otoliths (Suga and Nakahara, 2012).

Most of the genome-based studies of fishes have focused on growth traits, innate immunity, and/or sex determination (Cai et al., 2019; Zhou et al., 2019). However, no genome study related to the otolith growth and development has been reported yet. One of the main structural proteins in the organic matrix is Otolin-1a, also named as inner ear-specific collagen in some reports, containing calcium-binding sites; its C1q-like domain forms a stable trimer in calcium-containing solutions, suggesting that it participates in the correct arrangement of otolith to the inner ear sensory epithelium and may act as a nucleating agent for crystallization and stabilization of the otolith matrix (Murayama et al., 2005; Hołubowicz et al., 2017). Interestingly, we found that in the third collagen-like domain of Otol1a, the 319th amino acid is substituted by Ala (A), and both positions of 320 and 321 were lost in the family Sciaenidae.

SPARC, a major bone protein with an essential role for fish otolith normal growth and development (Kang et al., 2008), is multifunctional. It is able to bind both collagens and calcium. SPARCL1 and SPARCL2 were derived from SPARC through whole-genome duplication (WGD). When oc90 is absent, both sparc and sparcl1 mRNA levels were significantly upregulated to compensate for the lack of Oc90 and promoted biomineralization of murine otoconia (Xu et al., 2010). In our present study, the sequences of calcium-binding region in SPARC of various fishes showed high conservation in most sites; however, the position 274 of large yellow croaker and spiny head croaker is Asp (D), which might have a higher calcium-binding affinity at a high pH condition (Tang and Skibsted, 2016) and act as a crystal nucleation center whether directly binding with inorganic crystals or interacting with crystal binding proteins; in fact, these two modes are involved in the interaction between bone matrix protein and hydroxyapatite (or apatite; Xu et al., 2010). The PolyPhen-2 and PROVEAN results of amino acid substitution sites 65 and 204 showed possible functional changes of SPARC from the family Sciaenidae. All these data suggest that SPARC in family Sciaenidae plays an important role in calcium and collagen binding capacity, which may relate with formation of well-developed otoliths. While SPARCL1 in the family Sciaenidae was detected as a positive selected gene, whether its function in fish otoliths is similar to that in mice remains unclear. Verification by more studies is required.

Oc90 is a matrix protein of otolith with two PLA2L domains. Although these two domains do not possess enzymatic activity, they contain potential glycosylation sites, retain calcium-binding capacity, and provide a rigid structure for potential CaCO3 deposition (Petko et al., 2008). It is necessary for the early events of otolith biomineralization to play an important role in recruiting other proteins to form the organic matrix (Yang et al., 2011). In the Oc90 of spiny head croaker and large yellow croaker, several sites were substituted at PLA2L domains, which may lead to some changes in the calcium-binding capacity of this protein.

According to the substitutions or deletions of the abovementioned candidate gene sites that were identified at the genomic level, we speculate that these changes may have a relationship with otolith formation, resulting in the interesting status of well-developed otoliths in the family Sciaenidae.

## Conclusion

In this study, a high-quality chromosome-level genome assembly of spiny head croaker was constructed. Some amino acid substitutions or deletions in several otolith-related genes (such as substitutions in Oc90, Otol1a, SPARC, and deletions in Otol1a) were identified. These changes may be critical for well-developed otoliths in the family Sciaenidae. Our genome resources will provide genetic assistance for in-depth studies on detailed molecular mechanisms of the formation and development of well-developed otoliths in various Sciaenidae species.

## Data Availability

The original contributions presented in the study are publicly available in the CNGB Nucleotide Sequence Archive using accession number CNP0001197.
