# Supplementary material for: Whole-Genome Sequencing and Genome-Wide Studies of Spiny Head Croaker (Collichthys lucidus) Reveals Potential Insights for Well-Developed Otoliths in the Family Sciaenidae
Source: Front Genet. 2021 Sep 30;12:730255. doi: 10.3389/fgene.2021.730255 (PMC8515026; doi:10.3389/fgene.2021.730255)
Supplement: Supplementary file 1 [file DataSheet1.zip › FigureS10.SPARCL1_PolyPhen-2(protein and position of zebrafish as reference).pdf]

Protein Acc	Position	AA_1	AA_2	Description
sparcl1	212	S	A	N/A

Results

Prediction/Confidence

PolyPhen-2 v2.2.2r398

HumDiv

This mutation is predicted to be **POSSIBLY DAMAGING** with a score of **0.805** (sensitivity: **0.84**; specificity: **0.93**)

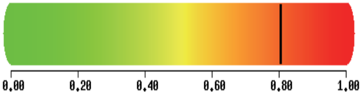

HumVar

This mutation is predicted to be **POSSIBLY DAMAGING** with a score of **0.478** (sensitivity: **0.83**; specificity: **0.81**)

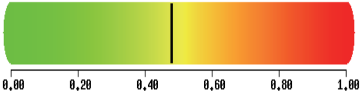

Details

Multiple sequence alignment

UniProtKB/UniRef100 Release 2011_12 (14-Dec-2011)

```
QUERY              EIDVEMMP--LESNVEKAAQQVAQTDEQGALFEESDG S AESEIPADLDYAADSGLLQALHTLTPAKHPNS---QLL
sp|UPI00017B1FA3#1 EVDVEMMP--LEGNVEKPAEEPAQTDERGALFEESDG S TESEIPADLDYAADSGLLQAPQTPTPPKQPDS---RLL
sp|UPI00022B4188#1 EVKEDSVEDVIEEKVQK-MTHSERKEEPTEALLEEDG S TESVIPVDLDYAADSSLLQPLQTASANDKTHSDDTKPT
sp|G3Q196#1        EVEVENVS--LKEKEEISAVKKADREEGGSLLEESDG S TESDIAVDVNYAADSGIVQPLQSASA---------KIK
sp|A4PE74#1        --------------LEKKVENPAGREEKVASPEETES S TESEIIADLDYAADSGVVRPLQTVSAEVITSAHEIQSI
sp|B3DJ32#1        ------------------------------------- - --------------------------------------
sp|Q4SDK1#1        ------------------------------------- - --------------------------------------
sp|UPI00017B1FA4#1 ------------------------------------- - --------------------------------------
sp|Q6PVV6#1        ------------------------------------- - --------------------------------------
sp|G1N6G1#1        ------------------------------------- - --------------------------------------
sp|E1C4U1#1        ------------------------------------- - --------------------------------------
sp|G3UR35#1        ------------------------------------- - --------------------------------------
sp|G1L936#1        ------------------------------------- - --------------------------------------
sp|UPI00022F6A7B#1 ------------------------------------- - --------------------------------------
sp|Q6P7A8#1        ------------------------------------- - --------------------------------------
sp|P24054#1        ------------------------------------- - --------------------------------------
sp|G3V7X5#1        ------------------------------------- - --------------------------------------
sp|P70663#1        ------------------------------------- - --------------------------------------
```

Shown are 75 amino acids surrounding the mutation position (marked with a black box). An interactive version of the complete alignment is also available.
