# Supplementary material for: Whole-Genome Sequencing and Genome-Wide Studies of Spiny Head Croaker (Collichthys lucidus) Reveals Potential Insights for Well-Developed Otoliths in the Family Sciaenidae
Source: Front Genet. 2021 Sep 30;12:730255. doi: 10.3389/fgene.2021.730255 (PMC8515026; doi:10.3389/fgene.2021.730255)
Supplement: Supplementary file 1 [file DataSheet1.zip › FigureS6.Otop1_multialign.pdf]

```
                                    10        20        30        40        50        60        70        80        90       100
zebrafish_otop1             MVEHGGTDSMWLNKYNPAAASSASSSSSSDAENKLFSRLKVSLTKKYPQKNAELLSAQYGTNLLLLGVSVMLALAAQSGPVKEEHLLSFITVLMLVQLVW  100
fugu_otop1                  MVEHNGLDIMCSNKY....CHSSSSSSSSEHDKKIFSKLKCNLSRDYPKKNAEIVSGQYGTNVLLVGAALMLAIAHHGPTVKEDHFLSFVTCLIIVQLFW   96
European_sea_base_otop1     MVEHSGLDIMCLNKY....CHSSSSSSSSEHDKKIFSKLKRSLSGDYPRKNAEILSGQYGTNVLLIGAALMLAIAHHGPSVKEEHLLSFVTCLMILQLIW   96
large_yellow_croaker_otop1  MVEHSGLDIMCLNKY....CHSSSSSSSSEHDKKVFSKLKQSLSGDYPRKNAEILSGQYGTNVLLIGASLMLAIAHHGPAVKEEHLLSFVTCLMILQLIW   96
spiny_head_croaker_otop1    MVEHSGLDIMCLNKY....CHSSSSSSSSEHDKKVFSKLKQSLSGDYPRKNAEILSGQYGTNVLLIGASLMLAIAHHGPAVKEEHLLSFVTCLMILQLIW   96
consensus                   !!!!*!!!!!!!!!!    !!!!!!!!!!!!!!! !!!!! !!!*!!!*!!!!!!!!!!!!!!!!*!!*!!!!!!!!!! !!!!!!!!!!!!!!!*!!*!
                                                                                     Transmembrane                Transmembrane

                                   110       120       130       140       150       160       170       180       190       200
zebrafish_otop1             MLCYMIRRERERSPVPERDAHAGASWIRGGLTMLALLSLIMDAFRIGYFVGYHSCISAALGVYPIVHALHAISQVHFLWFHIKDVIKKYETFERFGVIHA  200
fugu_otop1                  MMWYILVRHRRKDARTERDVHATSCWIRGGLTLLALLSLIMDAFRIGYYVGYHSCVSAVLGVYPVIHATHTVAQVHFLWFHIKDVIKSLETFERFGVIHA  196
European_sea_base_otop1     MMWYILVRDRQKNTRTEKDVHATTCWIRGGLTLLALLSLIMDAFRIGYYVGYQSCVSAVLGVYPVIHATHTIAQVHFLWFHIKDVIKSFETFERFGVIHA  196
large_yellow_croaker_otop1  MMWYILVRDRQKNSRTEKDVHATTCWIRGGLTLLALLSLIMDAFRIGYYVGYQSCVSAVLGVYPVIHATHTIAQVHFLWFHIKDVIKSFETFERFGVIHA  196
spiny_head_croaker_otop1    MMWYILVRDRQKNSRTEKDVHATTCWIRGGLTLLALLSLIMDAFRIGYYVGYQSCVSAVLGVYPVIHATHTIAQVHFLWFHIKDVIKSFETFERFGVIHA  196
consensus                   !!!!!!!!*!*!* !!!*!!!!!*!!!!!!!!!!!!!!!!!!!!!!!!!!!!*!!!!!!!!!!!!!!!!!!!!!!!!!!!!!!!!!!!*!!!!!!!!!!!
                                                     Transmembrane          Transmembrane    Transmembrane

                                   210       220       230       240       250       260       270       280       290       300
zebrafish_otop1             VFTNLLLWCNGVMSETEHFMHNHRRRLIEMGYANLSTVDVQPHCNCTTSVCSMFSTSLYYLYPFNIEYHIFVSAMLFVMWKNIGRTLDRHSNRKRRSTGT  300
fugu_otop1                  VFTNLLLWCNGVMSEAEHFLNNHMRRLSALGYANLTIVHAEPQCNCTTSTCSMFSSSLYYLYPFNIEYHIFVAATLFVMWKNIGRTIDLSSTRKRVATKT  296
European_sea_base_otop1     VFTNLLLWCNGVMSEAEHFLNNHKRRLSALGYGNLTIVHSEPHCNCTTSTCSMFSSSLYYLYPFNIEYHIFVSAMLFVMWQNIGRTIDLSSNRKRLATKT  296
large_yellow_croaker_otop1  VFTNLLLWCNGVMSEAEHFLNNHKRRLSALGYGNLTIVHSEPHCNCTTSTCSMFSSSLYYLYPFNIEYHIFVSAMLFVMWKNIGRTIDLSSNQKRMATKT  296
spiny_head_croaker_otop1    VFTNLLLWCNGVMSEAEHFLNNHKRRLSALGYGNLTIVHSEPHCNCTTSTCSMFSSSLYYLYPFNIEYHIFVSAMLFVMWKNIGRTIDLSSNQKRMATKT  296
consensus                   !!!!!!!!!!!!!!!!!!!!!!!*!!!!!!!!*!!!!!!*!!!!!!!!!!!!!!!!!!!!!!!!!!!!!!!!!!!!!!!!!!!!!!!!!!!!*!! !!!!
                              Transmembrane                                               Transmembrane

                                   310       320       330       340       350       360       370       380       390       400
zebrafish_otop1             TGLLLGPLGGLVALASSVSVLVVYLIHLEKTEEMHEAAVSMFYYYGVAMMACMCVGSGTGLLVYRMENRPMDTGSNPARTLDTELLLASSLGSWLMSWCS  400
fugu_otop1                  QGLTLGPILGLLALASTIGVLVVYITHMEESVRTRQSAISMFYIYGIVMLVFMSIGGAVGLLIYRADYLPLDTTKNPSRQLDTKLLFGSSVGSWLMSWCS  396
European_sea_base_otop1     QGLTLGPILGLLALASTIGILVVYITHVEESLKMRQSAISMFYIYGIIMLVFMCSAGTSGLLIYRADHIPLDTSKNPSRQLDTELLFGSSIGSWLMSWCS  396
large_yellow_croaker_otop1  QGLTLGPILGLLALASTIGILVVYITHVEESLRMRQSAISMFYIYGMVMLVFMCSASASGLLIYRADHMPLDTSKNPSRQLDTELLFGSSIGSWLMSWCS  396
spiny_head_croaker_otop1    QGLTLGPILGLLALASTIGILVVYITHVEESLRMRQSAISMFYIYGMVMLVFMCSASASGLLIYRADHMPLDTSKNPSRQLDTELLFGSSIGSWLMSWCS  396
consensus                   !!!!!!!!!!!!!!!!!!!*!!!!!!!*!!!**!!!!!!!!!!!!! *!!!!!!*****!!!!!!!!* !!!!*!!!!!!!!!!!!!!!!*!!!!!!!!!
                                    Transmembrane                             Transmembrane                        Transmembrane

                                     410       420       430       440           450          460        470             480
zebrafish_otop1             VVASVAEAGQKSPSFSWTSLTYSLLLVLEKCIQNLFIVESLYRRH....SEEEEDAAAPQVFSVA...VPPYDGILNHGYEAHDK.....HREAEPAAGS  488
fugu_otop1                  IVAVLGA..ESSPPYRWTNLIYSLLTVLEKYTQNLFIIESLYRQQVHTKRDDPELLPAPEIFSVTSSLAPPYTGIINRAYDTPDRACVAMENEQGESGQV  494
European_sea_base_otop1     IVAALGT..NSSPPYRWTNLVYSLLIVLEKYIQNLFIIESLYRQQEDGEREDPELPAAPEIFSVTSSLAPPYNGIINRAYETPDRACVTMENDQEESGQV  494
large_yellow_croaker_otop1  IVAVLGT..NSSPPYRWTNLIYSLLIVLEKYIQNLFIIESLYRQQEDTESEDPELPAAPEIFSVTSSLAPPYNGIINRAYETPDRTCVTMENEQEESGEV  494
spiny_head_croaker_otop1    IVAVLGT..NSSPPYRWTNLIYSLLIVLEKYIQNLFIIESLYRQQEDTESEDPELPAAPEIFSVTSSLAPPYNGIINRAYETPDRTCVTMENEQEESGEV  494
consensus                   !!!*!!*  *!!!!!!!!!!*!!!!*!!!!!!!!!!!!!!!!!!!*****!!!!!*!!!!!!!!!!!!!!!!*!!!!!!!!!!!! !!*!!!!!!!!! !

                            490       500       510       520       530       540       550       560       570       580
zebrafish_otop1             HALSRKQPDAPLPAGQRLDVTPGRKRQILKNICMFLFMCNISLWILPAFGCRPQYDNPLENETFGTSVWTTVLNVAIPLNLFYRMHSVASLFEVFRKV  586
fugu_otop1                  YKCPIKPSEVSLPGGNKVVRPLNVKKQVLKNISIFLVMCNISLWILPAFGCRPQYDNGLEQETFGFSIWTTVLNFAIPLNLFYRMHAVSSLFEVFCQV  592
European_sea_base_otop1     YRCPRKPSEVPLPVGNKVVEPPNIKRQILKNIAVFLLMCNISLWILPAFGCRPQYDNGLEQETFGFSIWTTVLNFAIPLNLFYRMHSVASLFEVFRRV  592
large_yellow_croaker_otop1  YRCPRKPSEVPLPVGNKVVDPPNIKRQILKNIAVFLLMCNISLWILPAFGCRPQYDNGLEQETFGFSIWTTVLNFAIPLNLFYRMHSVASLFEVFRRV  592
spiny_head_croaker_otop1    YRCPRKPSEVPLPVGNKVVDPPNIKRQILKNIAVFLLMCNISLWILPAFGCRPQYDNGLEQETFGFSIWTTVLNFAIPLNLFYRMHSVASLFEVFRRV  592
consensus                   !*!!!!!!!!!!!*!!!!! !!!*!!!!!!!!**!!*!!!!!!!!!!!!!!!!!!!!!!!!!!!!!!!!!!!!!!!!!!!!!!!!!!!!!!!!!!!*!
                                                            Transmembrane                Transmembrane
```

X non conserved

X similar

X $\geq$50% conserved

X $\geq$80% conserved
