# Supplementary material for: Whole-Genome Sequencing and Genome-Wide Studies of Spiny Head Croaker (Collichthys lucidus) Reveals Potential Insights for Well-Developed Otoliths in the Family Sciaenidae
Source: Front Genet. 2021 Sep 30;12:730255. doi: 10.3389/fgene.2021.730255 (PMC8515026; doi:10.3389/fgene.2021.730255)
Supplement: Supplementary file 1 [file DataSheet1.zip › FigureS9.SPARCL1_multialign.pdf]

```
zebrafish_sparcl1             MKKEVFLFCFLVTALAAYVQSKPHGKK...QHIKHIATPKE..QSESVKLHGSMDGVLPTFLPFESSTQ...DEDTSEETESRVEMNQAE   82
fugu_sparcl1                  MRACCVFLYLLAATFALSVKSKPHGKH...HKTLNVAKDKDIIAEEANEPQ.....ALPTLLPLEESGTEQEVEDLSSEDNANANKEIRG   82
European_sea_bass_sparcl1     MRACLVFLCLLSATFALSVKSKPHGKHHGLHKTSHTAKEKDIITEEANKPQ.....ILPTLVPFEASSQEQEDEELSSEDNANANKEEGE   85
large_yellow_croaker_sparcl1  MRACLVFLCLLAATFALSVKSKPHGKHGGLHKTSHTAKEKDIVTEEANKPQ.....ILPTLVPLEVSSHEQEDEESSSDDNANANKED..   83
spiny_head_croaker_sparcl1    MRACLVFLCLLAATFALSVKSKPHGKHGGLHKTSHTAKEKDIVTEEANKPQ.....ILPTLVPLEVSSHEQEDEESSTDDNANASKED..   83
consensus                     !!!!*!!!!!!*!!!!!!!!!!!!!!! **!!!*!*!!*!!! *!!!!!!!     *!!!!*!*! !* !!!!!* !**!!!!!*!! * 
```

```
zebrafish_sparcl1             AEEPEGSDGGEKKEPLLLSDEALTQLLQEPVEEDEDDHSESLEKDE.V............................MDK...VEQSTEDG  140
fugu_sparcl1                  IKMPKRSS...KTEAVLMSDEELTELLKKE...EQEVEERELEEEEEAEVEEGKEQSVEISEDSEM......LNDQLDREDAEEGAEGGE  160
European_sea_bass_sparcl1     VEVTERSD...KTTAFLLGEDELVIILKKEAEKEQEAEERALEEEE.AKTDKGKVESGEIIEDEEAEVEEKSLDDKSEM...EEEVTEE.  167
large_yellow_croaker_sparcl1  FEVTEGSD...KSTAVLLSEEELVDLLKKEAEEEQEAEEIVLEEEE.VKTEKSEEESGEIIEDE...VEEKTLDDKLER...EEDAKEEE  163
spiny_head_croaker_sparcl1    FEVTEGSD...KSTAVLLSEEELVDLLKKEAEEEQEAEEIVLEEEE.VKTEKSEEESGEISEDE...VEEKTLDDKLEI...EEDAEEEE  163
consensus                      !**!*!!   ! *!*!!!*!!!* !!!!!*!*!!!*!!  !!!!! *****  **!*!! !!*    **** !*!***   !! * !**
```

```
zebrafish_sparcl1             ESKTETENEAE...............SKEEDS........KD......TDSSTESEIPMDLDYTTDRDASQPLPI....KLDEA...KVL  194
fugu_sparcl1                  EIIDEDGEDAEMLDKEEIDVEMMPLESNVEKAAQQVAQTDEQGALFEESDGSAESEIPADLDYAADSGLLQALHTLTPAKHPNS...QLL  247
European_sea_bass_sparcl1     EVKEESEEDEEMLEKEEVRVEMMAPEKKEEKSVEKIADREEE......TDGSTESEVPVDLDYAADSSVVQPLHILSAKIKPHADDMEPL  251
large_yellow_croaker_sparcl1  EEKEESVEDAELLEKEEV.............EVEKI.DREEQ........GAFLEETGLDLDYAADSGILSPLQL...EKTPHTDDTQP.  227
spiny_head_croaker_sparcl1    EEKEESVEDAELLEKEEV.............EVEKI.DREEQ........GAFLEETGLDLDYAADSGILSPLQL...EKTPHTDDTQP.  227
consensus                     ! !*!* !!!! !*!!!*           *  **** ***!*       *!* **! * !!!!!!!!* **!!      ! !* ** ***
```

```
zebrafish_sparcl1             TTME....DTVEDVIPTATASYESQQSNSDIAEEQEELEDQAQEELKEPENDIEKGKNDQQDEKNI..LEPGI.....SE..........  263
fugu_sparcl1                  PETEGNEQKTSEEELPAASDD......SAE.AADGGEMSEHDE.....................................DQQATSKDEQ  293
European_sea_bass_sparcl1     SKTDFKDKET..............NVQNTE.AVDSDEVSDQDQDEDSEAKSK.EKSSAKKEPEANVDPQEPSSNTEFESG..........  315
large_yellow_croaker_sparcl1  ...DVKEKETSEKGLQTIADDYEQNVQNTE.AVDSEEVSDQDQ...............................................  266
spiny_head_croaker_sparcl1    ...DVKEKETSEKGPQTIADDYEQNVQNTE.AVDSEEASDQDQ...............................................  266
consensus                        * ****!*!    *  **** ***!*! !*!**! !!!!!
```

```
zebrafish_sparcl1             ...................GKMAKKD..KKKNESKSNGKGKGKKQRNSELLEKMQSDQAGAIEDTEELAQKKDQEKVTEATENTESKP.R  331
fugu_sparcl1                  GQELDGVTPDPKESADVPMSKMGGKEEEKSR....RHSKRKTKKQQRNHMVGK.HFPQSEQPQSGQEQSQ...QESESSSAENTGSKAKR  375
European_sea_bass_sparcl1     ...................SRGTGREEEKSKNDSGSHTKGKTRKQKKNQRARK.HSPQRDETQSGQEQGQQDPQESEGSSTDNTVHKAKR  385
large_yellow_croaker_sparcl1  ...................TRVAGKDEEKSKNDSGSQTKGKTRKQKKNQRARK.HSPQSEETQTGQEQSQQDPQESEGSSTDNTVQKAKR  336
spiny_head_croaker_sparcl1    ...................TRAAGKDEEKSKNDSGSQTKGKTRKQKKNQRARK.HSPQSEETQTGQEQSQQDPQESEGSSTDNTAQKAKR  336
consensus                                         * *!!*!!!!!!*!*! *!!!!*!!**!****! !!!!****! !!!!*!***!!!!*!!!*!!  !!!!
```

```
zebrafish_sparcl1             KKNGKWTRLVGMNPVQIRATMDLYPDVR..LTRHSNGQGVPADPCENFRCKRGKTCKLNDENKPLCVCQEPTECPPNVNDFEHVCGTDNK  419
fugu_sparcl1                  RRAGKWGSIVGMNPVQIRATVDLYPSTRSALAGHVHHPEAPADPCTNFPCKRGKTCKLDADNKPGCVCQQASECPPSVNDFDRVCGTDNK  465
European_sea_bass_sparcl1     RRAGKWGPLVGMNPVQIRATADLYPSSRSSLGGGVHHPEAPTDPCDNFPCKRGKTCKLDADNNPGCVCQAPSECPRSVNEFDHVCGTDNK  475
large_yellow_croaker_sparcl1  RRAGKWGPLVGMNPVQIRATADLYPSSRSSPSS...HPDAPADPCDNFPCKRGKTCKLDAENKPGCVCQEPSECPPGLNEFDHVCGTDNK  423
spiny_head_croaker_sparcl1    RRAGKWGPLVGMNPVQIRATVDLYPNSRSSPSG...HPDAPADPCDNFPCKRGKTCKLDADNKPGCVCQEPSKCPPGLNEFDHVCGTDNK  423
consensus                     !!!!!!!*!!!!!!!!!!!! !!!!**!!** *   !!  !!!!!!*!!!!!!!!!!!!!!*!!!!!!!!*!!!!!! *!*!!!!!!!!!!
```

Follistatin-like domain

Kazal-like domain

```
zebrafish_sparcl1             TYDSSCHLFATKCGLEGTKLGHRLHLDYTGSCKFIAPCVESELVQFPLRMRDWLKNVLLQLYEHDSMSPGFLTAKQRIRVQKIYESERRL  509
fugu_sparcl1                  TYDSSCQLFAAKCNLEGTKRGHRLHLDYTGPCKLIPSCLDAELVQFPLRMRDWLKNVLLQLYDHDSTAPGFLTAKQRFKVKKIFESERRL  555
European_sea_bass_sparcl1     TYETSCELFATKCNLEGTKRGHRLHLDYTGSCKFIPPCVDTELIQFPLRMRDWLKNVLLQLYEHDSMSPGFLTPKQRFRVKKIFESERRL  565
large_yellow_croaker_sparcl1  TYDTACELFATKCNLEGTKRGHRLHLDYTGSCKLIPACEDSELVQFPLRMRDWLKNVLLQLYEHDSVSSGFLTPKQRFRVKKIFESERRL  513
spiny_head_croaker_sparcl1    TYDTSCELFATKCNLEGTKRGHRLHLDYTGSCKLIPACEDSELVQFPLRMRDWLKNVLLQLYEHDSVSSGFLTPKQRFRVKKIFESERRL  513
consensus                     !!!*!!*!!!!!!!!!!!!!!!!!!!!!!!!!*!!  !  !*!!!!!!!!!!!!!!!!!!!!!!!!! !*!!!!*!!!!!!!!!!!!!!!!
```

SPARC Ca bdg

Kazal-like domain

Calcium binding region

```
zebrafish_sparcl1             HAGDHPVEILQQDFEKNYNMYIYPVHWQFAQMDQHPSDRFLTHSELAPLRVPLVPMEHCTSVFFQMCDADKDKLVSFKEWCSCFGIKEED  599
fugu_sparcl1                  HAGNHSAELLAQDFEKNYNMYIYPVHWQFAQLDQHPSDRFLSHSELAPLRVPLVPMEHCTSRFFQECDADKDKQVSFKEWTSCFGIKNED  645
European_sea_bass_sparcl1     HAGDHSVELLAQDFEKNYNMYIYPVHWQFAQLDQHPSDRFLSHSELAPLRVPLVPMEHCTSRFFQECDADKDKQVSFKEWTSCFGIKNED  655
large_yellow_croaker_sparcl1  HAGDHSVELLVQDFEKNYNMYIYPVHWQFAQLDQHPADRFLTHSELAPLRVPLVPMEHCTSRFFQECDADKDKLVSFKEWTSCFGIKNED  603
spiny_head_croaker_sparcl1    HAGDHSVELLVQDFEKNYNMYIYPVHWQFAQLDQHPADRFLTHSELAPLRVPLVPMEHCTSRFFQECDADKDKLVSFKEWTSCFGIKNED  603
consensus                     !!!!!!!!!! !!!!!!!!!!!!!!!!!!!!!!!!!*!!!!*!!!!!!!!!!!!!!!!!!!!!!!!!!!!!!!*!!!!!!!!!!!!!!!!
```

SPARC Ca bdg

EF hand

```
zebrafish_sparcl1             MNTNLLF   606
fugu_sparcl1                  MDVNQLF   652
European_sea_bass_sparcl1     MDVNQLF   662
large_yellow_croaker_sparcl1  TDVNLLF   610
spiny_head_croaker_sparcl1    TDVNLLF   610
consensus                     *!!!*!!
```

X non conserved
X similar
X ≥50% conserved
X ≥80% conserved
