# Supplementary material for: Whole-Genome Sequencing and Genome-Wide Studies of Spiny Head Croaker (Collichthys lucidus) Reveals Potential Insights for Well-Developed Otoliths in the Family Sciaenidae
Source: Front Genet. 2021 Sep 30;12:730255. doi: 10.3389/fgene.2021.730255 (PMC8515026; doi:10.3389/fgene.2021.730255)
Supplement: Supplementary file 1 [file DataSheet1.zip › supplementary materials.docx]

**Some scripts used in analysis**

1. Assembly
2. platanus assemble -t 14 -k 27 -m 300 -s 3 -f shortreads_fq1.gz shortreads_fq2.gz 2 -o raw > platanus.contig.log
3. DBG2OLC Contigs raw_contig.fa LD 0 K 17 KmerCovTh 4 MinOverlap 30 AdaptiveTh 0.01 RemoveChimera 1 f pbreads.fa
4. Reduce redundant sequences
5. Python redundans.py -v -f pilon_genome.fa -i short_reads.fq1.gz short_reads.fq2.gz –log run.log -o outdir -t 6 --identity 0.85 --overlap 0.36 --minLength 1000 --noscaffolding --nogapclosing –resume
6. Annotation
7. Repetitive annotation: The Repetitive annotation was performed according to the manuals and protocols provided by the developer of the corresponding bioinformatics tools.
8. PASA:

PASApipeline-v2.3.3//misc_utilities/accession_extractor.pl <pb_isoseq_hq.fa> pasa/FL_accs.txt

Launch_PASA_pipeline.pl -c alignAssembly.config -t pb_isoseq_hq.fa -C -R -g final.fa --ALIGNERS blat,gmap --CPU 8 -N 200 -f FL_accs.txt

Launch_PASA_pipeline.pl -c annotCompare.config -g chromosome.fa -t ISOseq.fasta.clean -A -L --CPU 8 –annots forpasa.gff3

3) Functional annotation: The Repetitive annotation was performed according to the manuals and protocols provided by the developer of the corresponding bioinformatics tools.

4. Genome evolution

1) TreeFam method:

1. blastall -p blastp -m8 -e 1e-7 -F F -d all.pep.fa -i all.pep.fa -o all_vs_all.blast.m8
2. solar.pl -a prot2prot -f m8 -z all_vs_all.blast.m8 > all_vs_all.solar.raw ; convert bit score to percent score with “hcluster_score = bit2raw(bitscore) / bit2raw(best_bitscore) * 100” to output all_vs_all.s olar.forHC.
3. hcluster_sg -w 10 -s 0.34 -m 500 -b 0.1 -C category.txt.genes all_vs_all.s olar.forHC > all_vs_all.hcluster; get single-copy families from all_vs_all.hcluster
4. muscle -in genefamily.cds.fa -out genefamily.cds.muscle ; muscle -in genefamily.pep.fa -out genefamily.pep.muscle; merge all muscle result of single-copy families in each species to super-gene sequences.
5. phylogenetic tree:

raxmlHPC-SSE3 -p 12345 -m GTRGAMMA -s single-copy.cds.phy.phase1 -n single-copy.tree -f a -o L.episosteus -x 12345 -# 1000

1. Divergence: baseml4.9 baseml.ctl; mcmctree4.9 mcmctree.ctl;
2. Gene family expansion and contraction: The Repetitive annotation was performed according to the manuals and protocols provided by the developer of the corresponding bioinformatics tools.
3. Identify positive selection genes:
   1. prank -quiet -protein -d=single-copy_gene_family*.fa -o=./prank_out ; convert prank alignments to CDS alignments.
   2. cd single-copy_gene_family*; paml-4.9e/bin/codeml single-copy_gene_family*.null.ctl; paml-4.9e/bin/codeml single-copy_gene_family*.alt.ctl

5. otolith related genes

1) blastall -p tblastn -d chromosome.fa -i refgene.fa -e 1e-5 -m 8 -F F -o gene.blast

2) solar.pl -a prot2genome2 -f m8 gene.blast > gene.blast.solar

3) Genewise: The analysis was performed according to the manuals and protocols provided by the developer of the corresponding bioinformatics tools.
