# Supplementary figures and images for: Whole-Genome Sequencing and Genome-Wide Studies of Spiny Head Croaker (Collichthys lucidus) Reveals Potential Insights for Well-Developed Otoliths in the Family Sciaenidae
Source: Front Genet. 2021 Sep 30;12:730255. doi: 10.3389/fgene.2021.730255 (PMC8515026; doi:10.3389/fgene.2021.730255)

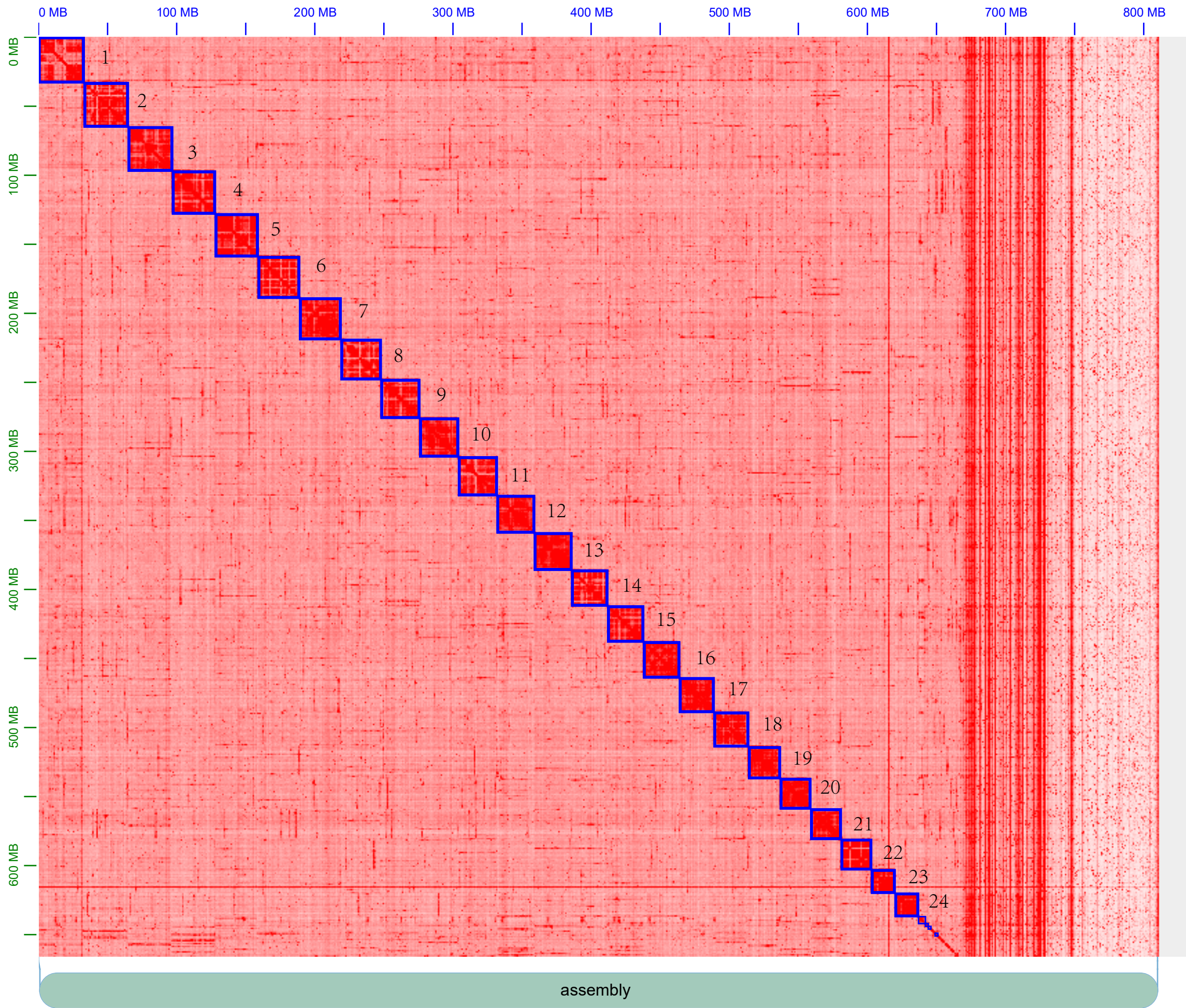

Supplement: Supplementary file 1 [file DataSheet1.zip › Figure S1 .The chromosomes contact map based on Hi-C data.pdf]

### Distribution of exon length

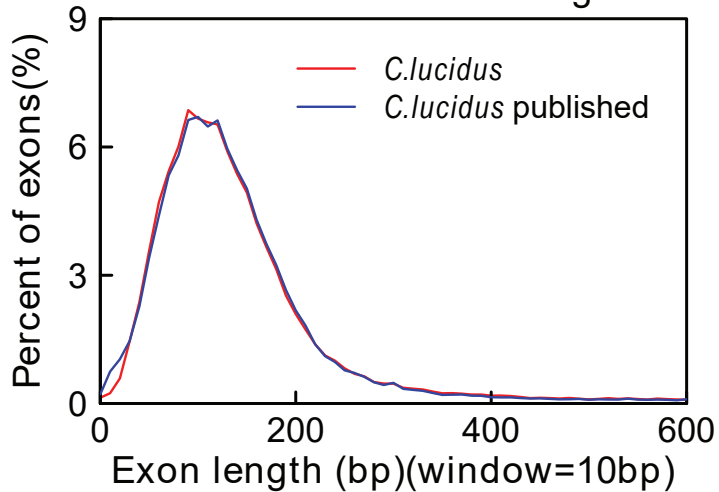

### Distribution of intron length

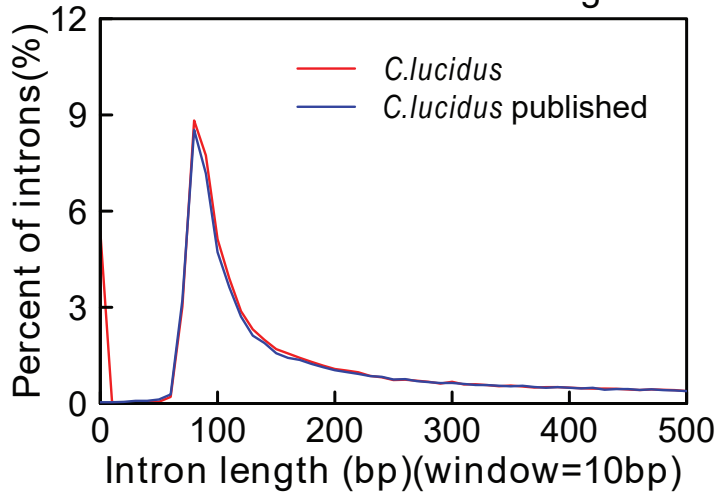

Supplement: Supplementary file 1 [file DataSheet1.zip › FigureS2.Distribution of exon and intron length between our data and published geneset.pdf]

C. lucidus

L. crocea

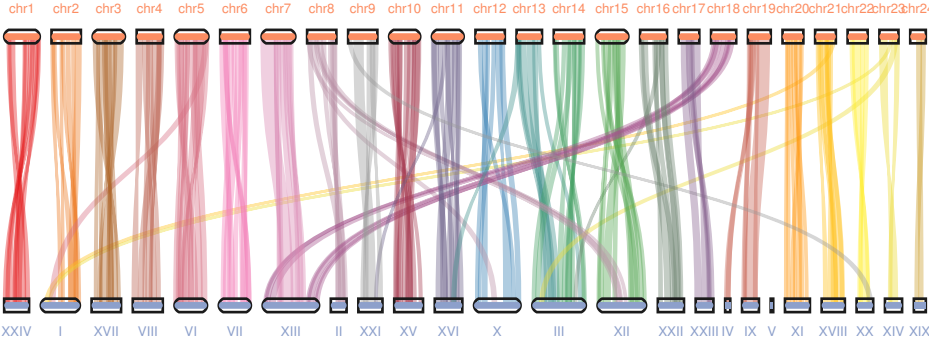

Supplement: Supplementary file 1 [file DataSheet1.zip › FigureS3. Synteny between spiny head croaker and large yellow croaker.pdf]
