# Supplementary material for: Whole-Genome Sequencing and Genome-Wide Studies of Spiny Head Croaker (Collichthys lucidus) Reveals Potential Insights for Well-Developed Otoliths in the Family Sciaenidae
Source: Front Genet. 2021 Sep 30;12:730255. doi: 10.3389/fgene.2021.730255 (PMC8515026; doi:10.3389/fgene.2021.730255)
Supplement: Supplementary file 1 [file FigureS11.SPARCL2_multialign.pdf › FigureS11.SPARCL2_multialign.pdf]

```
                                     10        20        30        40        50        60        70        80        90
zebrafish_sparcl2              MQL.LAQLFFLTAMLHLGVV..MGGRAQRKQRQAEEKLRPYLGRVDPESLCQLLKCHSPIGSWCQVVKEEGVLTPKCVCPSTCPRQRAPVCSVLGKTYSN   97
fugu_sparcl2                   .........................RGQHKQTQAEENLRPYIGRVDPEKLXELLRCHSPVGSWCQVIQKNRVRIPKYVCPQSCPSQGTPVCSVLGNTYAN   75
European_sea_bass_sparcl2      MNLTFALFLLLS..LHICKA..MGSRAQRKQRQAEESLRPYIGRVEPEKLCELLKCHSPVGSWCQVVRENGVLIPKCVCPQSCPRQRAPVCSVLGRTYGN   96
large_yellow_croaker_sparcl2   MDLNLALFLFLS..LHTCTATSQGGRAQRKQRQAEESLRPYIGRVEPEKLCELLKCHSPVGSWCQVVQENGLFVPKCVCPQSCPRQRAPVCSVLGRTYGN   98
spiny_head_croaker_sparcl2     MDLNLALFLFLS..LHTCTATSQGGRAQRKQRQAEESLRPYIGRVEPEKLCELLKCHSPVGSWCQVVQENGVFVPKCVCPQSCPRQRAPVCSVLGRTYGN   98
consensus                      !  ! *!****!*  !! * *   !*!!!!!!!!!!!*!!!!!!!!*!!!!!!!!!!!!!!!!!!!!!!*!!!!  !!!!!!!!!!!!!!!!!!!!!!*!!*!
                                           SPARC Ca bdg
                                                                                                  Kazal-like domain

                                                                                                                 Calcium binding region
                               100       110       120       130       140       150       160       170       180       190
zebrafish_sparcl2              ECLLHKEACRKERRIGKAHNGACLVSET...GCSEEEFGQFPYRLL..DWFLLLSRMGERYTPAAPSQSCLSHTQRMQLAQRRFVLLDRNRDGKLSRRDL   192
fugu_sparcl2                   ECVLHREACRKRCRIGLAHRGACLGSYTELINTT....NPSLYALLFPNWFLLLSRVGKSYSPGASSQRCLSHRQRTQLAQERFTMLDRNKDGTLSRVDL   171
European_sea_bass_sparcl2      ECLLHKEACRKRRRTGLAHTGPCLVPKA...KCTEEELSQFPYRLL..DWFLLLSRMGESYAPAAPPQNCLSHAQRTQLAQQRFTILDKNKDGKLSRRDL   191
large_yellow_croaker_sparcl2   ECLLHKEACRKRRRNGLAHTGPCLVPKA...NCTEEEFNQFPYRLL..DWFLLLSRMGESYAPAAPPQSCLSHAQRAELAQKRFTVLDKNKDGKLSLRDL   193
spiny_head_croaker_sparcl2     ECLLHKEACRKRRRNGLAHTGPCLVPKA...NCTEEEFNQFPYRLL..DWFLLLSRMGESYAPAAPPQSCLSHTQREELAQKRFSTLDKNKDGKLSLRDL   193
consensus                      !!!!!!!!!!!!!! !!!!*!*!!!***   *!!!!!**!!!!!!!  !!!!!!!!!!!!!*!!!!*!*!!!!*!! *!!! !!* !!*!!!!!!!*!!!
                                           SPARC Ca bdg
                               Kazal-like domain                                                             EF hand

                                         Calcium binding region
                                     200       210       220       230       240       250
zebrafish_sparcl2              RKLHYKRMPLEHCAQRFFQSCDKNQNKKVTLREWTSCLVKRSERWFEDFMS.MKMGSPKLCP.VQDNNL   259
fugu_sparcl2                   KKMTYRTMPLESCAMLFFQSCDHNRNKKVTLQQXTTCLVDRTEAWFHSFMCYMRMGSQKLCPEVKN...   237
European_sea_bass_sparcl2      RKLRYKRMPLEHCATPFFQSCDRNRNRKVTLQEWTTCLVDRSEAWFYHFMS.VRMGSRKLCPTIKENVL   259
large_yellow_croaker_sparcl2   RKLRYKRMPLEHCAAPFFQSCDRNRNRKVTLREWTTCLVDRTEAWFFHFMS.MKMGSRKLCPTNKTNHL   261
spiny_head_croaker_sparcl2     RKLRYKRMPLEHCAAPFFQSCDRNRNRKVTLREWTTCLVDRTEAWFFHFMS.MKMGSRKLCPTNKANHL   261
consensus                      !!!*!!!!!!!!!! *!!!!!!*!!!*!!!!*!!!!!!!!!*!!!!  *!!!  !*!!!*!!!!*  !  !  !
                                   SPARC Ca bdg
                               EF hand       EF hand
```

X non conserved

X similar

X $\geq 50\%$ conserved

X $\geq 80\%$ conserved
