# Supplementary material for: Whole-Genome Sequencing and Genome-Wide Studies of Spiny Head Croaker (Collichthys lucidus) Reveals Potential Insights for Well-Developed Otoliths in the Family Sciaenidae
Source: Front Genet. 2021 Sep 30;12:730255. doi: 10.3389/fgene.2021.730255 (PMC8515026; doi:10.3389/fgene.2021.730255)
Supplement: Supplementary file 1 [file FigureS4.Oc90_multialign.pdf › FigureS4.Oc90_multialign.pdf]

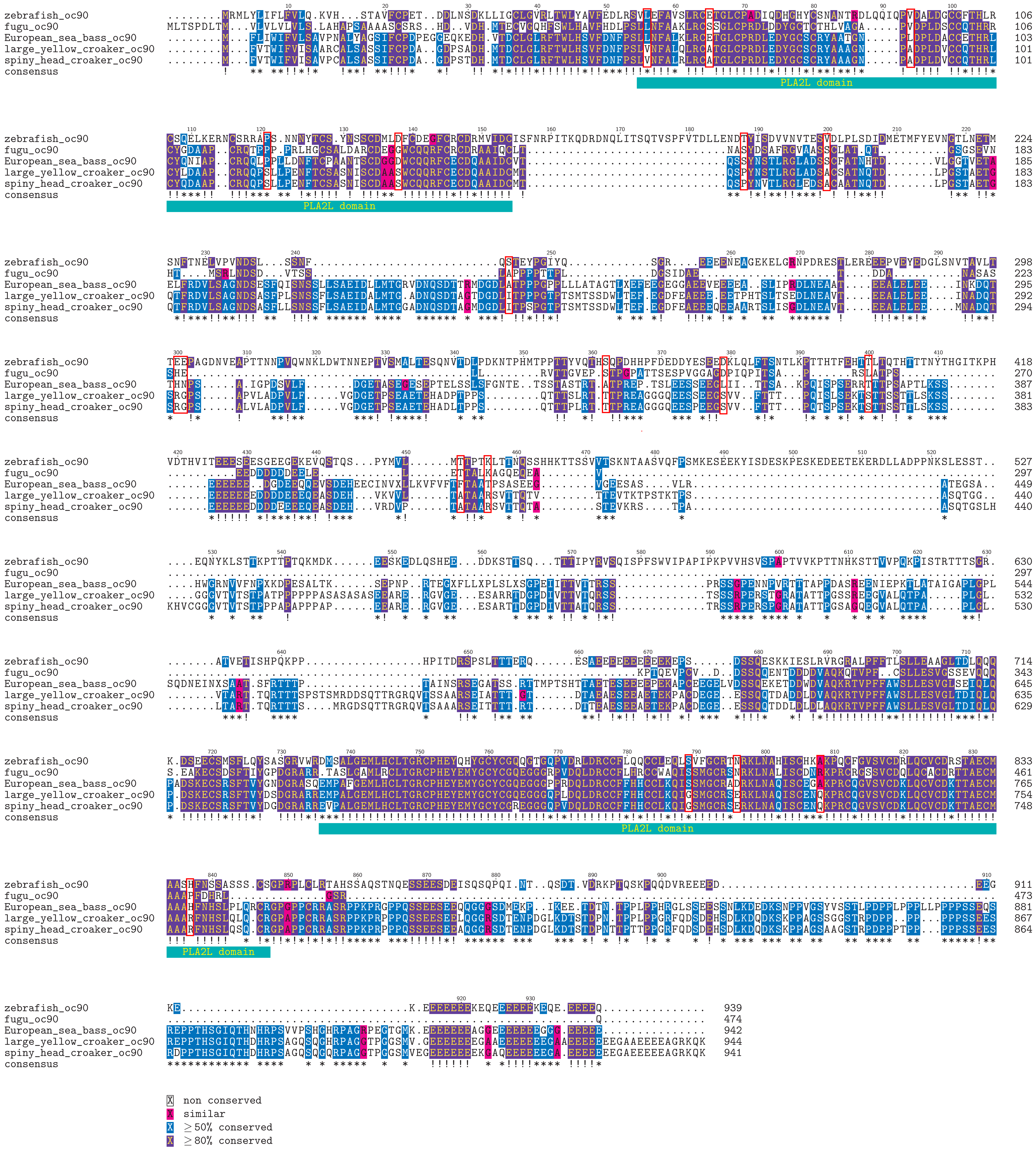
zebrafish_oc90
fugu_oc90
European_sea_bass_oc90
large_yellow_croaker_oc90
spiny_head_croaker_oc90
consensus
PLA2L domain
non conserved
similar
≥ 50% conserved
≥ 80% conserved
