# Supplementary material for: Whole-Genome Sequencing and Genome-Wide Studies of Spiny Head Croaker (Collichthys lucidus) Reveals Potential Insights for Well-Developed Otoliths in the Family Sciaenidae
Source: Front Genet. 2021 Sep 30;12:730255. doi: 10.3389/fgene.2021.730255 (PMC8515026; doi:10.3389/fgene.2021.730255)
Supplement: Supplementary file 1 [file FigureS5.OMP1_multialign.pdf › FigureS5.OMP1_multialign.pdf]

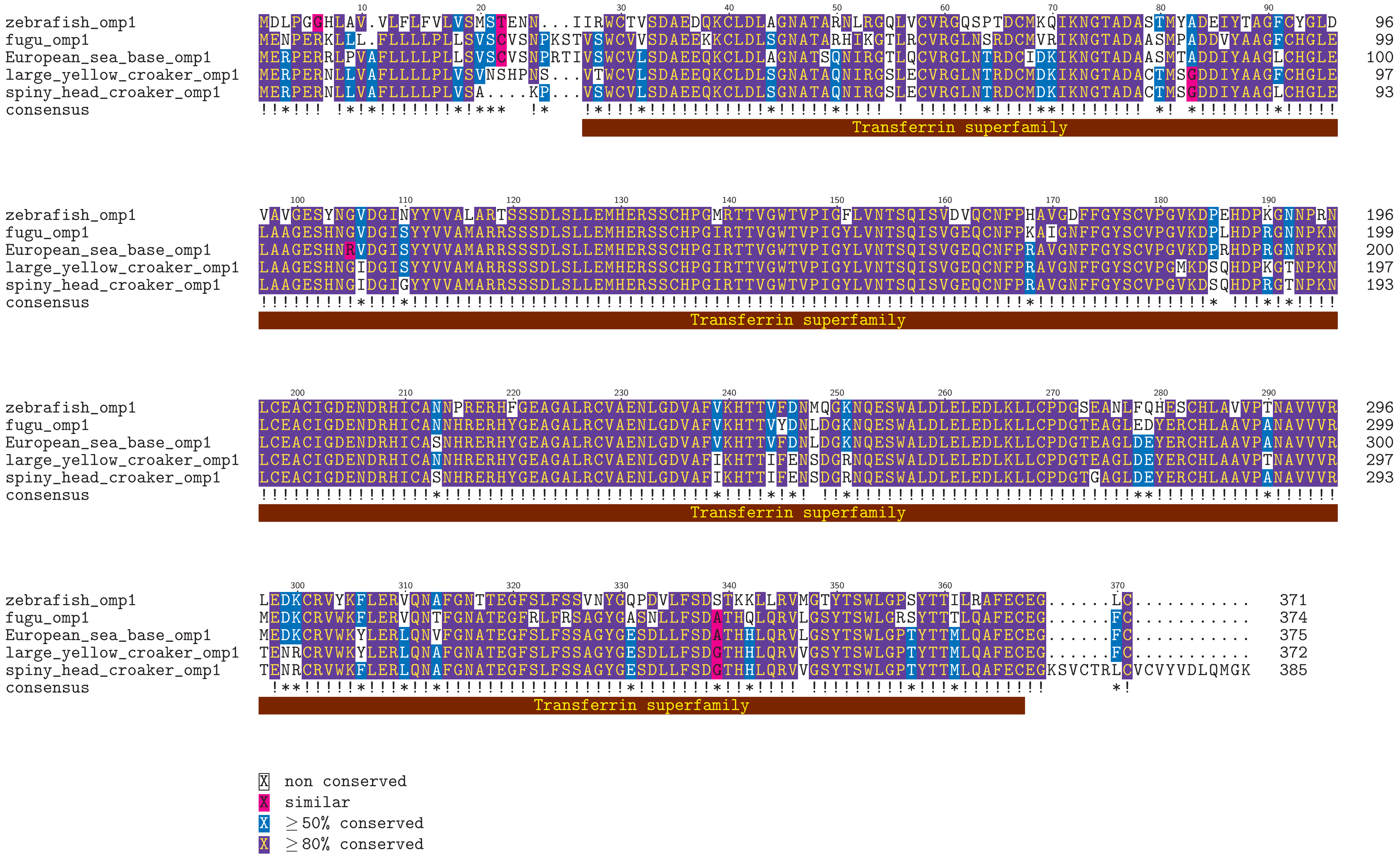
zebrafish_omp1
fugu_omp1
European_sea_base_omp1
large_yellow_croaker_omp1
spiny_head_croaker_omp1
consensus
96
99
100
97
93
196
199
200
197
193
296
299
300
297
293
371
374
375
372
385
Transferrin superfamily
non conserved
similar
≥50% conserved
≥80% conserved
