# Supplementary material for: Whole-Genome Sequencing and Genome-Wide Studies of Spiny Head Croaker (Collichthys lucidus) Reveals Potential Insights for Well-Developed Otoliths in the Family Sciaenidae
Source: Front Genet. 2021 Sep 30;12:730255. doi: 10.3389/fgene.2021.730255 (PMC8515026; doi:10.3389/fgene.2021.730255)
Supplement: Supplementary file 1 [file FigureS7.Otol1a_multialign.pdf › FigureS7.Otol1a_multialign.pdf]

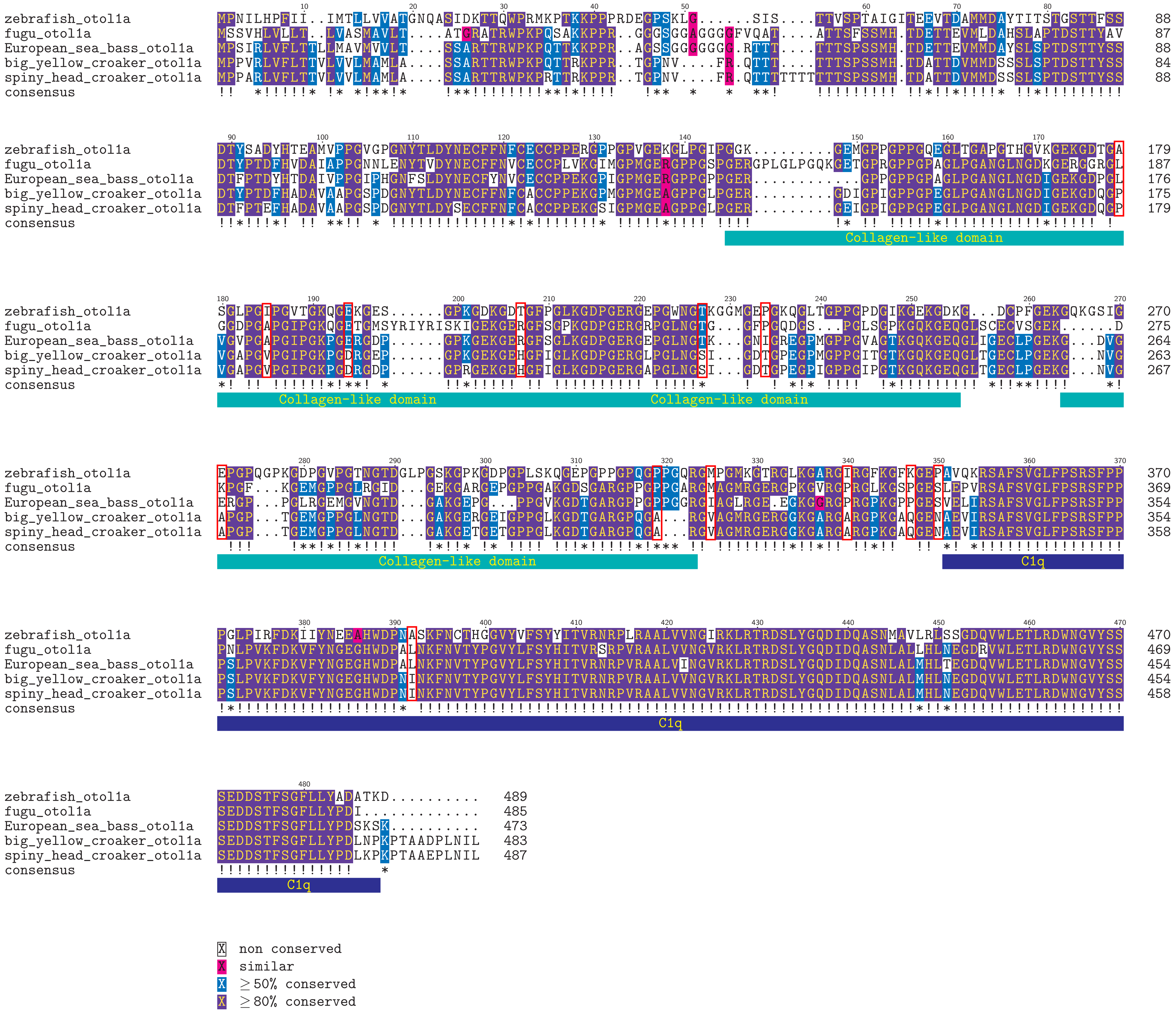
zebrafish_otol1a MPNILHPFII..IMTLLVVATGNQASIDKTTQWPRMKPTKKPPPRDEGPSKLG......SIS....TTVSPTAIGITEEVTDAMMDAYTITSTGSTTFSS 88
fugu_otol1a MSSVHLVLLT..LVASMAVLT....ATGRATRWPKPQSAKKPPR..GGGSGGAGGGGFVQAT....ATTSFSSMH.TDETTEVMLDAHSLAPTDSTTYAV 87
European_sea_bass_otol1a MPSIRLVFLTTLLMAVMVVLT....SSARTTRWPKPQTTKKPPR..AGSSGGGGGGG.RTTT....TTTSPSSMH.TDETTEVMMDAYSLSPTDSTTYSS 88
big_yellow_croaker_otol1a MPPVRLVFLTTVLVVLMAMLA....SSARTTRWPKPQTTRKPPR..TGPNV....FR.QTTT....TTTSPSSMH.TDATTDVMMDSSSLSPTDSTTYSS 84
spiny_head_croaker_otol1a MPPARLVFLTTVLVVLMAMLA....SSARTTRWPKPRTTRKPPR..TGPNV....FR.QTTTTTTTTTTSPSSMH.TDATTDVMMDSSSLSPTDSTTYSS 88
consensus !! *!!!!!* !* *!**!* !**!!!!!!!!!**!*!!!! !** * * **! !!!!!!!!! !!*!!*!!!!* !!*!!!!!!!!!
zebrafish_otol1a DTYSADYHTEAMVPPGVGPGNYTLDYNECFFNFCECCPPERGPPGPVGEKGLPGIPGGK.........GEMGPPGPPGQEGLTGAPGTHGVKGEKGDTGA 179
fugu_otol1a DTYPTDFHVDAIAPPGNNLENYTVDYNECFFNVCECCPLVKGIMGPMGERGPPGSPGERGPLGLPGQKGETGPRGPPGPAGLPGANGLNGDKGERGRGL 187
European_sea_bass_otol1a DTFPTDYHTDAIVPPGIPHGNFSLDYNECFYNVCECCPPEKGPIGPMGERGPPGPPGER...........GPPGPPGPAGLPGANGLNGDIGEKGDPGL 176
big_yellow_croaker_otol1a DTYPTDFHADAVAAPGSPDGNYTLDYNECFFNFCACCPPEKGPMGPMGEAGPPGLPGER.........GDIGPIGPPGPEGLPGANGLNGDIGEKGDQGP 175
spiny_head_croaker_otol1a DTFPTEFHADAVAAPGSPDGNYTLDYSECFFNFCACCPPEKGSIGPMGEAGPPGLPGER.........GEIGPIGPPGPEGLPGANGLNGDIGEKGDQGP 179
consensus !!*!!!*! !! **!! * !!!!!!!!!!!!!*!*!!!!!!!!* !!!!!*!!!! !!!! !* !! !!!!!*!!!!!!!!!!!!!*!!!!! !
Collagen-like domain
zebrafish_otol1a SGLPGIPGVTGKQGEKGES......GPKGDKGDTGFPGLKGDPGERGEPGWNGTKGGMGEPGKQGLTGPPGPDGIKGEKGDKG...DCPFGEKGQKGSIG 270
fugu_otol1a GGDPGAPGIPGKQGETGMSYRIYRISKIGEKGERGFSGPKGDPGERGRPGLNGTG...GFPGQDGS...PGLSGPKGQKGEQGLSCECVSGEK......D 275
European_sea_bass_otol1a VGVPGAPGIPGKPGERGDP......GPKGEKGERGFSGLKGDPGERGEPGLNGTK...GNIGREGPMGPPGVAGTKGQKGEQGLIGECLPGEKG...DVG 264
big_yellow_croaker_otol1a VGAPGVPGIPGKPGDRGEP......GPKGEKGEHGFIGLKGDPGERGLPGLNGSI...GDTGPEGPMGPPGITGTKGQKGEQGLTGECLPGEKG...NVG 263
spiny_head_croaker_otol1a VGAPGVPGIPGKPGDRGDP......GPRGEKGEHGFIGLKGDPGERGAPGLNGSI...GDTGPEGPIGPPGIPGTKGQKGEQGLTGECLPGEKG...NVG 267
consensus *! !! !!!!!!*!**! * !!*!!!!! !! !!!!!!!!!! !!!!!* ! ! *!* !!!! !*!!!!!!!!! *!!**!!!! *!
Collagen-like domain
Collagen-like domain
zebrafish_otol1a EPGPQGPKGDPGVPGTNGTDGLPGSKGPKGDPGPLSKQGEPGPPGPQGPPGQRGMPGMKGTRGLKGARGIRGFKGFKGEPAVQKRSAFSVGLFPSRSFPP 370
fugu_otol1a KPGF...KGEMGPPGLRGID...GEKGARGEPGPPGAKGDSGARGPPGPPGARGMAGMRGERGPKGVRGPRGLKGSPGESLEPVRSAFSVGLFPSRSFPP 369
European_sea_bass_otol1a ERGP...PGLRGEMGVNGTD...GAKGEPG...PPGVKGDTGARGPPGPPGGRGIAGLRGE.EGKGGRGPRGPKGPPGESVELIRSAFSVGLFPSRSFPP 354
big_yellow_croaker_otol1a APGP...TGEMGPPGLNGTD...GAKGERGEIGPPGLKGDTGARGPQGA...RGVAGMRGERGGKGARGARGPKGAQGENAEVIRSAFSVGLFPSRSFPP 354
spiny_head_croaker_otol1a APGP...TGEMGPPGLNGTD...GAKGETGETGPPGLKGDTGARGPQGA...RGVAGMRGERGGKGARGARGPKGAQGENAEVIRSAFSVGLFPSRSFPP 358
consensus !!! !**!*!!*!!!! !*!!* !* !!!! !!!*!!!!!*!*** !! !!!!!!!!*!!*!! !!*!! !! *! *!!!!!!!!!!!!!!!!!
Collagen-like domain
C1q
zebrafish_otol1a PGLPIRFDKIIYNEEAHWDPNASKFNCTHGGVYVFSYYITVRNRPLRAALVVNGIRKLRTRDSLYGQDIDQASNMAVLRLSSGDQVWLETLRDWNGVYSS 470
fugu_otol1a PNLPVKFDKVFYNGEGHWDPALNKFNVTYPGVYLFSYHITVRSRPVRAALVVNGVRKLRTRDSLYGQDIDQASNLALLHLNEGDRVWLETLRDWNGVYSS 469
European_sea_bass_otol1a PSLPVKFDKVFYNGEGHWDPALNKFNVTYPGVYLFSYHITVRNRPVRAALVINGVRKLRTRDSLYGQDIDQASNLALMHLTEGDQVWLETLRDWNGVYSS 454
big_yellow_croaker_otol1a PSLPVKFDKVFYNGEGHWDPNINKFNVTYPGVYLFSYHITVRNRPVRAALVVNGVRKLRTRDSLYGQDIDQASNLALMHLNEGDQVWLETLRDWNGVYSS 454
spiny_head_croaker_otol1a PSLPVKFDKVFYNGEGHWDPNINKFNVTYPGVYLFSYHITVRNRPVRAALVVNGVRKLRTRDSLYGQDIDQASNLALMHLNEGDQVWLETLRDWNGVYSS 458
consensus !*!!!!!!!!!!!!!!!!!!* !!!*!!*!!!!!!!!!!!!!!!!!!!
C1q
zebrafish_otol1a SEDDSTFSGFLLYADATKD.......... 489
fugu_otol1a SEDDSTFSGFLLYPDI............. 485
European_sea_bass_otol1a SEDDSTFSGFLLYPDSKSK.......... 473
big_yellow_croaker_otol1a SEDDSTFSGFLLYPDLNPKPTAADPLNIL 483
spiny_head_croaker_otol1a SEDDSTFSGFLLYPDLKPKPTAAEPLNIL 487
consensus !!!!!!!!!!!!!!! *
C1q
X non conserved
X similar
X ≥ 50% conserved
X ≥ 80% conserved
