# Supplementary material for: Whole-Genome Sequencing and Genome-Wide Studies of Spiny Head Croaker (Collichthys lucidus) Reveals Potential Insights for Well-Developed Otoliths in the Family Sciaenidae
Source: Front Genet. 2021 Sep 30;12:730255. doi: 10.3389/fgene.2021.730255 (PMC8515026; doi:10.3389/fgene.2021.730255)
Supplement: Supplementary file 1 [file FigureS8.Otol1b_multialign.pdf › FigureS8.Otol1b_multialign.pdf]

```
                                        10         20         30         40         50         60         70         80         90
zebrafish_otol1b             MLSSLLFLDRQKMGMFCARSVLVTVLALTF.IILCDTFKPTQRPKYQYTKKP.PREVVQT..TTYAGKPTVTARIVDYTKTREQRTPVYVTESTTVTADS   96
fugu_otol1b                  ............MRN.TRQLILVVVAVVALNTGHCVEAKTTQKPKYQYTKKPVPQVTVQSPLTTSAGK...THKLVPTAKSVQPH.PPPATEKPMYASHR   83
European_sea_bass_otol1b     ............MRIISCQSTLLAVVVVTLTVVSFAEAKTTQKPKYQYTKKPVPQITVHNPVTTSMPK...THKIVPSAKPVAPH.PLPITERTTYPSHV   84
big_yellow_croaker_otol1b    ............MRIISCQSTLVVIVVVTLTVVCCVEAKTTPKPKYQYTKKPVSQITVHSPVTTGMPK...THKVVPSTKPVEPR.PLPTTDKTTHPSHV   84
spiny_head_croaker_otol1b    ............MRIISCQSTLVAIVVVTLTVVCCVEAKTTPKPKYQYTKKPVPQITVHNPVTTGMPK...THKVVPSAKPVEPR.PLPTTEKTTHPSHV   84
consensus                                !!****!!*!! **!!!!*** !*!!!!!*!!!!!!!!!!!!!*!!* !*!! **!    !!! !!**!*!*!* !*! !!*!! *!!*

                              100        110        120        130        140        150        160        170        180        190
zebrafish_otol1b             YIDYPTDTSASPTAAKDNYTLDYNECYFNFCECCPPEKGPQGFKGDMGLPGPPGEKGAPGPSGLPGPIGPKGNSGSKGDKGEKGDQGNTGFAGTPGNQGK  196
fugu_otol1b                  FQNDYQPSTEAPGGGLENYTLDYNECYFNFCECCPPERGPRGPKGDRGLEGPPGERGPSGPAGLPGPTGMSGPLGLKGDKGERGERGGSGSAGQTGLPGK  183
European_sea_bass_otol1b     FPQYYTESPEAPGVGPENYTLDYNECYFNFCECCPPERGPRGPKGDRGLAGPPGERGFTGPAGLPGQPGLSGPMGLKGDKGEKGDRGGSGTAGSPGVPGK  184
big_yellow_croaker_otol1b    FPKYYTDITEAPGVGPENYTLDYNECYFNFCECCPPERGPRGPKGDRGLAGPPGDRGLTGAAGLPGSPGVSGPSGLKGDKGDKGDRGGSGTSGSPGGPGK  184
spiny_head_croaker_otol1b    FPKYYTDITEAPGVGPENYTLDYNECYFNFCECCPPERGPRGPKGDRGLAGPPGDRGLTGAAGLPGSPGVSGPIGLKGDKGDKGDRGGSGASGSPGGLGK  184
consensus                    !* !!!* *!!!!*!*!!!!!!!!!!!!!!!!!!!!!!!!!!!!!!!!*!!!!*!! *!*!!!!! *! !!! !!!!!!!*!!!!!!!! *!*!! *!!
                                                                                     [Collagen-like domain]

                              200        210        220        230        240        250        260        270        280        290
zebrafish_otol1b             AGMKGEMGNKGEKGAAGLPGFKGEKGEKGDPNFNVSKGDQGEPGKDGLPGPQGSA...GEKGEKGERGECGLLGDRGQKGEPGDPGSPGVRGDPGPSGQH  293
fugu_otol1b                  PGEKGDAGSKGEKGVMGLQGLQGDNGEKGEPGQNGTTGEKGEPGKEGP................GDKGECSTFGERGQKGEQGD............PGLN  255
European_sea_bass_otol1b     PGQKGDVGSKGEKGEIGLQGVKGGIGEKGEPGQNGTAGEKGEPGKEGPAGPPGVAVELGPKGDKGDKGECGIFGERGQKGERGDSGPPGIPGAMGIPGIN  284
big_yellow_croaker_otol1b    PGQKGDLGSKGEKGDIGLQGVKGGIGEKGEPGLNGTAGEKGEPGKEGPAGPPGMAIALGPKGDKGDKGECGLFGERGMKGEPGDPGPPGIPGVMGIPGIN  284
spiny_head_croaker_otol1b    PGQKGDLGSKGEKGDIGLQGVKGGIGEKGEPGLNGTAGEKGEPGKEGPAGPPGMAIALGPKGDKGDKGECGLFGERGMKGEPGDPGPPGIPGVMGIPGIH  284
consensus                    !!*!!! !!!!!!! *!!!!*!!**!!!!!!! !!!*!!!!!!!!!!!*!!*! !  *!*!!*!!!!!!!!*!!!!!*!!!*!!*!*!!**! *!*!!**
                                          [Collagen-like domain]                          [Collagen-like domain]

                                  300        310        320        330        340        350        360        370        380        390
zebrafish_otol1b             GMHGNPGITGERGEPGTPGPKGEPGIQGPPGTKGLRGLRGIKGDRGPQGKRGDRGLRGMKGAMGTSDSRIRSAFSVGLYPSKSFPPSGFPVRFDKVFYNG  393
fugu_otol1b                  GKHGSPGPGGVRGDPGPAGQRGEPGVRGAEGLQGVRGLFGPKGDRGYPGKKGERGFRGLKGAKGAQITQKRSAFSVGISPRKSFPPSGFPIRFDKIFYNE  355
European_sea_bass_otol1b     GKHGAPGPMGVRGDLGPTGQQGEPGVRGSQGPQGIRGVMGPKGDRGYPGMRGERGFRGFKGAKGSGVPQKRSAFSVGISPRKSFPPSGFPIRFDKVFYNE  384
big_yellow_croaker_otol1b    GKHGSSGPGGIRGDQGPPGPQGEPGVRGVQGPQGIRGMPGPKGDRGNPGKRGDRGFRGFKGAKGSGVPQTRSAFSVGISPRKSFPPSGFPIRFDKVFYNE  384
spiny_head_croaker_otol1b    GKHGSSGPGGVRGDQGPPGPQGEPGVRGLQGPQGIRGMPGPKGDRGNPGKRGDRGFRGVKGAKGSGVPQTRSAFSVGISPRKSFPPSGVPIRFDKVFYNE  384
consensus                    !!!!**!!*!*!!! !!*!**!!!!!!! *!*!!*!!  !!!!!!! !!!!!*!!!!! !!!!!****! !!!!!!!!!!!!!!!!!!!!!!!!!!!!!!
                                [Collagen-like domain]                                        [C1q]

                                 400        410        420        430        440        450        460        470        480        490
zebrafish_otol1b             ENHYDVVSCKFNCSYSGVYVFSYQITVRNKPLRASLVVNGVRKVRSRDTLQPQDIDQASNLVILKLDVGDQVWVETLRDWNGVYSSSEDDSTFSGFLLYP  493
fugu_otol1b                  ENHYNFTSNSFMCVHAGVYVFSFHITVRNQPLRATLVVNGSRKVRTRDSLYGQDIDQASTLVVLRLVVGDQVWLETFRDWNGVYASSEDDSIFSGFLLYS  455
European_sea_bass_otol1b     DNHFNVTSNSFTCVHAGVYVFSFHITVRNQPLRATLVVNGSRRVRTRDSLYGQDIDQASTLVVLRLAVGDQVWMETFRDWNGAYASSEDDSIFSGFLLYS  484
big_yellow_croaker_otol1b    ENHFNVTSNSFTCVHPGVYVFSFHITVRNQPLRATLVVNGSRRVRTRDSLYGQDIDQASTLVVLRLAAGDQVWMETFRDWNGAYASSEDDSIFSGFLLYS  484
spiny_head_croaker_otol1b    ENHFNVTSNSFTCVHPGVYVFSFHITVRNQPLRATLVVNGSRRVRTRDSLYGQDIDQASTLVVLRLAAGDQVWMETFRDWNGAYASNEDDSIFSGFLLYS  484
consensus                    !!!*!!!!!!!*!!! !!!!!!!!!!!!!!!!!!!!!!!!!*!!!!!!!!!!!!!!!!!!!!!!!!!**!!!!!*!!!!!!!!*!!!!!!!!!!!!!!!!
                                                                      [C1q]

zebrafish_otol1b             D..  494
fugu_otol1b                  EEA  458
European_sea_bass_otol1b     DNA  487
big_yellow_croaker_otol1b    DNV  487
spiny_head_croaker_otol1b    DNV  487
consensus                    !*
```

X non conserved

X similar

X ≥ 50% conserved

X ≥ 80% conserved
